# Level of Attention to Motherese Speech as an Early Marker of Autism Spectrum Disorder

**DOI:** 10.1001/jamanetworkopen.2022.55125

**Published:** 2023-02-08

**Authors:** Karen Pierce, Teresa H. Wen, Javad Zahiri, Charlene Andreason, Eric Courchesne, Cynthia C. Barnes, Linda Lopez, Steven J. Arias, Ahtziry Esquivel, Amanda Cheng

**Affiliations:** 1 Autism Center of Excellence, Department of Neurosciences, University of California San Diego, La Jolla

## Abstract

**Importance:**

Caregivers have long captured the attention of their infants by speaking in motherese, a playful speech style characterized by heightened affect. Reduced attention to motherese in toddlers with autism spectrum disorder (ASD) may be a contributor to downstream language and social challenges and could be diagnostically revealing.

**Objective:**

To investigate whether attention toward motherese speech can be used as a diagnostic classifier of ASD and is associated with language and social ability.

**Design, Setting, and Participants:**

This diagnostic study included toddlers aged 12 to 48 months, spanning ASD and non-ASD diagnostic groups, at a research center. Data were collected from February 2018 to April 2021 and analyzed from April 2021 to March 2022.

**Exposures:**

Gaze-contingent eye-tracking test.

**Main Outcomes and Measures:**

Using gaze-contingent eye tracking wherein the location of a toddler’s fixation triggered a specific movie file, toddlers participated in 1 or more 1-minute eye-tracking tests designed to quantify attention to motherese speech, including motherese vs traffic (ie, noisy vehicles on a highway) and motherese vs techno (ie, abstract shapes with music). Toddlers were also diagnostically and psychometrically evaluated by psychologists. Levels of fixation within motherese and nonmotherese movies and mean number of saccades per second were calculated. Receiver operating characteristic (ROC) curves were used to evaluate optimal fixation cutoff values and associated sensitivity, specificity, positive predictive value (PPV), and negative predictive value. Within the ASD group, toddlers were stratified based on low, middle, or high levels of interest in motherese speech, and associations with social and language abilities were examined.

**Results:**

A total of 653 toddlers were included (mean [SD] age, 26.45 [8.37] months; 480 males [73.51%]). Unlike toddlers without ASD, who almost uniformly attended to motherese speech with a median level of 82.25% and 80.75% across the 2 tests, among toddlers with ASD, there was a wide range, spanning 0% to 100%. Both the traffic and techno paradigms were effective diagnostic classifiers, with large between-group effect sizes (eg, ASD vs typical development: Cohen *d*, 1.0 in the techno paradigm). Across both paradigms, a cutoff value of 30% or less fixation on motherese resulted in an area under the ROC curve (AUC) of 0.733 (95% CI, 0.693-0.773) and 0.761 (95% CI, 0.717-0.804), respectively; specificity of 98% (95% CI, 95%-99%) and 96% (95% CI, 92%-98%), respectively; and PPV of 94% (95% CI, 86%-98%). Reflective of heterogeneity and expected subtypes in ASD, sensitivity was lower at 18% (95% CI, 14%-22%) and 29% (95% CI, 24%-34%), respectively. Combining metrics increased the AUC to 0.841 (95% CI, 0.805-0.877). Toddlers with ASD who showed the lowest levels of attention to motherese speech had weaker social and language abilities.

**Conclusions and Relevance:**

In this diagnostic study, a subset of toddlers showed low levels of attention toward motherese speech. When a cutoff level of 30% or less fixation on motherese speech was used, toddlers in this range were diagnostically classified as having ASD with high accuracy. Insight into which toddlers show unusually low levels of attention to motherese may be beneficial not only for early ASD diagnosis and prognosis but also as a possible therapeutic target.

## Key Points

**Question:**

Can levels of attention toward motherese speech, indexed through eye tracking, be used as a diagnostic marker of autism spectrum disorder (ASD), and are they associated with social and language abilities?

**Findings:**

In this diagnostic study of 653 toddlers aged 12 to 48 months, levels of fixation on motherese speech were high in toddlers without ASD but significantly reduced in toddlers with ASD. If a toddler fixated on motherese speech at or below 30%, the probability of that toddler being accurately identified as ASD was 94% and also signified an association with reduced social and language abilities.

**Meaning:**

The findings suggest that levels of attention to motherese speech may be a diagnostic and prognostic marker of ASD.

## Teaser

This diagnostic study investigates whether attention to high-affect motherese speech can be used as a diagnostic marker of autism spectrum disorder and language and social ability among toddlers aged 12 to 48 months.

## Introduction

Caregivers have long captured the attention of their infants and toddlers by speaking in motherese, a playful speech style characterized by exaggerated intonation contours, simple grammar, high pitch, and slow tempo, also known as parentese or infant-directed speech.^1^ Infants prefer to listen to this speech style relative to adult-directed speech from the first days of life,^2^ a preference ubiquitous across cultural and geographical boundaries,^3^ suggesting a possible genetic basis.^4^ Studies have noted that this highly affective speech style stimulates joint attention and learning and is associated with improved language acquisition, affective engagement, and emotional reactivity.^1,5,6^

It has long been known that children with autism spectrum disorder (ASD) show unusual and sometimes absent responses to auditory information in their environment. For example, children may fail to respond when their name is called^7,8^ or exhibit a poor understanding of word meanings.^9,10^ At the neural level, studies commonly report significantly reduced functional brain activity^10,11,12,13^ or delayed timing in response to speech sounds.^14,15,16^ Using behavioral techniques such as turning the head toward sounds emitted from a tape recorder, Kuhl and colleagues^17^ showed that 70% of children with ASD preferred to listen to computer-generated sounds over motherese, a finding that has been replicated.^18,19^ Yet, reduced response to motherese speech is not commonly reported among children with non-ASD delays, suggesting possible specificity for ASD and utility as a diagnostic marker.^20^

In studies of typically and atypically developing children, associations between exposure to motherese speech and early word recognition,^5^ mean length of utterance,^21^ and receptive language ability^22^ have been reported. Given the experience-dependent mechanisms that support learning during the first years of life,^23,24,25,26^ infants who do not pay attention to motherese speech—or human speech in general—would likely have more impaired language ability than children who pay close attention to social speech. In a brain imaging study, toddlers with ASD who showed the lowest levels of attention to motherese speech also showed the lowest levels of neural functional activation in speech-processing regions and lower language abilities than toddlers who paid greater attention to motherese speech.^27^

Eye tracking is a relatively new approach for rapidly and accurately characterizing attention in ASD^28,29,30,31,32,33,34,35,36,37^ and holds promise as a screening and diagnostic tool.^31,33^ Eye-tracking studies, however, have been largely restricted to the visual domain and have demonstrated reduced attention to social visual images in ASD.^30,38,39,40,41,42,43,44^ Although there are some exceptions,^45^ few studies have leveraged eye-tracking technology to understand attention patterns within the auditory domain, particularly in response to speech sounds. In theory, this could be achieved through gaze-contingent technology wherein the location where a toddler looks on a monitor triggers specific speech and nonspeech sounds.

While visual attention is commonly indexed as a percent fixation metric (eg, percent fixation time on faces), other eye-tracking metrics, such as saccades, may also provide useful information. Unique saccade profiles can be associated with levels of anxiety^46,47^ but are not necessarily correlated with overall fixation quantities. For example, studies have shown dissociations between saccade and fixation levels in ASD, with some toddlers with ASD who exhibit high levels of fixation on nonsocial images also showing low saccade rates.^31,32^ Examining saccade metrics in conjunction with traditional percent fixation metrics could provide new insight regarding how toddlers with ASD visually and auditorily examine their world. This may be essential for improving the diagnostic classification accuracy of eye-tracking tests and help to uncover unique biological subtypes of ASD. The aim of this study was to investigate whether level of attention toward motherese speech can be used as a diagnostic classifier of ASD and is associated with language and social ability.

## Methods

This diagnostic study was approved by the institutional review board at the University of California San Diego (UCSD). Parents gave written informed consent, and all testing occurred at the UCSD Autism Center. The study followed the Standards for Reporting of Diagnostic Accuracy (STARD) reporting guideline (eMethods in Supplement 1). Race and ethnicity information was obtained via parent report and was included as required for federal grants; toddlers’ race and ethnicity were categorized per National Institutes of Health definitions. Categories for race were African American/Black, American Indian/Alaska Native, Asian, Native Hawaiian/Pacific Islander, White, more than 1 race, and not reported, unknown, or other. Ethnicity was categorized as Hispanic or Latino, not Hispanic or Latino, and unknown or not reported.

### Participants and Clinical Testing

Toddlers were recruited through 2 mechanisms: community referrals (eg, website) or a general population-based screening method called Get SET Early,^48^ using the Communication and Symbolic Behavior Scales Developmental Profile Infant-Toddler Checklist,^49,50^ that results in the detection of ASD as young as 12 months.^48,51^ Toddlers younger than 30 months who were referred were invited for a reevaluation every 9 to 12 months until their third birthday, when a final diagnosis was given.

Toddlers aged 12 to 48 months participated in 1 or more motherese eye-tracking tests: motherese vs traffic (ie, noisy, moving vehicles on a highway), motherese vs techno (ie, moving shapes with musical sounds), and motherese vs flat affect. Across all tests, 11% of the sample was excluded for a range of reasons such as inattention to the video. Details regarding recruitment, exclusion, and testing overlap are given in the eMethods and eFigures 1 to 3 in Supplement 1.

Toddlers and their parents participated in a series of tests including the Autism Diagnostic Observation Schedule (ADOS),^52^ the Mullen Scales of Early Learning,^53^ and the Vineland Adaptive Behavior Scales.^54^ All assessments were administered by ADOS-certified, licensed clinical psychologists (C.C.B. and S.J.A.) blind to eye-tracking results.

Based on diagnoses given at the final diagnosis age, toddlers were categorized into 5 contrast groups (ASD, ASD features, delay, typical development [TD], and typical sibling of an ASD proband). Details regarding diagnostic criteria and clinical scores are given in the Table and eFigure 4 and the eMethods in Supplement 1.

**Table.** Clinical Characteristics of Toddlers Who Completed 1 or More Motherese Eye-Tracking Tests

Characteristic	Toddlers (N = 653)^a^				
	ASD (n = 422)	ASD features (n = 29)	Delay or other (n = 75)	TD (n = 115)	TypSibASD (n = 12)
Sex					
Female	100 (23.70)	9 (31.03)	26 (34.67)	34 (29.57)	4 (33.33)
Male	322 (76.30)	20 (68.97)	49 (65.33)	81 (70.43)	8 (66.67)
Age, mean (SD), mo					
Motherese vs traffic	26.71 (7.38)	26.55 (9.54)	22.21 (8.67)	25.44 (8.52)	22.33 (9.85)
Motherese vs techno	26.94 (7.81)	27.04 (9.08)	24.07 (9.93)	25.35 (8.51)	23.33 (11.45)
Motherese vs flat affect	28.01 (8.66)	29.26 (8.81)	24.13 (10.04)	28.21 (8.15)	23.13 (9.69)
Ethnicity					
Hispanic or Latino	169 (40.05)	10 (34.48)	30 (40.00)	15 (13.04)	1 (8.33)
Not Hispanic or Latino	219 (51.89)	17 (58.62)	37 (49.33)	86 (74.78)	10 (83.33)
Unknown or not reported	34 (8.06)	2 (6.90)	8 (10.67)	14 (12.17)	1 (8.33)
Race					
African American/Black	7 (1.66)	1 (3.45)	1 (1.33)	6 (5.22)	0
American Indian/Alaska Native	5 (1.18)	1 (3.45)	2 (2.67)	0	0
Asian	69 (16.35)	0	5 (6.67)	8 (6.96)	0
Native Hawaiian/Pacific Islander	3 (0.71)	1 (3.45)	1 (1.33)	0	0
White	218 (51.66)	19 (65.52)	46 (61.33)	72 (62.61)	10 (83.33)
>1 Race	45 (10.66)	4 (13.79)	3 (4.00)	14 (12.17)	1 (8.33)
Not reported, unknown, or other^b^	75 (17.77)	3 (10.34)	17 (22.67)	15 (13.04)	1 (8.33)
Mullen T score, mean (SD)^53^					
Visual reception	34.73 (13.33)	50.62 (11.00)	45.19 (9.96)	55.23 (11.03)	52.33 (7.75)
Fine motor	34.91 (17.67)	43.93 (7.67)	44.75 (11.45)	51.90 (8.73)	50.00 (9.28)
Receptive language	29.10 (15.15)	44.86 (8.89)	40.55 (11.83)	51.33 (10.99)	54.83 (16.57)
Expressive language	28.00 (16.06)	46.28 (9.25)	34.20 (10.85)	51.17 (10.24)	57.58 (10.15)
Early learning composite	67.47 (21.04)	93.24 (11.05)	83.04 (14.34)	104.80 (14.61)	109.42 (13.23)
Vineland, mean (SD)^54^					
Communication	77.45 (16.67)	94.34 (9.44)	87.56 (12.96)	100.55 (9.26)	100.00 (7.21)
Daily living	82.24 (13.22)	92.97 (12.73)	92.11 (10.83)	96.77 (11.42)	99.08 (8.98)
Socialization	81.80 (13.64)	91.72 (14.52)	90.67 (12.00)	100.23 (9.69)	100.42 (7.43)
Motor skills	90.66 (11.45)	97.41 (13.75)	94.12 (11.66)	99.31 (9.62)	100.36 (4.59)
Adaptive behavior composite	79.21 (11.60)	91.72 (11.13)	88.03 (11.23)	98.19 (10.38)	98.83 (8.22)
ADOS score, mean (SD)^52^					
SA/CoSo	13.70 (4.31)	5.21 (3.72)	3.24 (2.29)	2.67 (1.94)	2.83 (1.27)
RRB	5.68 (1.75)	2.90 (1.47)	1.72 (1.28)	1.37 (1.21)	0.75 (1.06)
Total	19.39 (5.09)	8.10 (3.72)	4.96 (2.51)	4.04 (2.21)	3.58 (1.98)

Abbreviations: ADOS, Autism Diagnostic Observation Schedule; ASD, autism spectrum disorder; RRB, restricted and repetitive behavior; SA/CoSo, social affect and communication total score; TD, typical development; TypSibASD, typical sibling of an ASD proband.

^a^ Data are presented as the number (percentage) of toddlers unless otherwise indicated.

^b^ Other was not broken down further.

#### Apparatus and Procedure

Either a Tobii T120 (365 toddlers [55.90%]) or Tobii Pro Spectrum 600 Hz (288 toddlers [44.10%]) eye tracker in combination with E-Prime software, version 3.0 (Psychology Software Tools), was used to create gaze-contingent paradigms wherein the *x*-*y* coordinate of a toddler’s fixation triggered the appearance of a specific movie file. More details are given in the eMethods and eFigure 5 in Supplement 1.

#### Dog-Cat Gaze-Contingent Preexperiment Training

Toddlers were introduced to the concept of gaze contingency prior to the experiment. Details are given in the eMethods and eFigure 6 in Supplement 1.

#### Motherese Paradigms

Toddlers were presented with a series of 3 paradigms designed to quantify interest in motherese speech. Each of the paradigms consisted of 2 movies placed side by side, with 1 side always containing an actress speaking in a motherese style, using speech that contained high rates of questions, inflections, and emotional prosody.^1,55^ In the motherese vs traffic paradigm, the contrasting movie depicted cars moving on a busy highway with accompanying car horns and traffic noise, while in the motherese vs techno paradigm, the contrasting movie contained moving abstract shapes and numbers and was accompanied by musical chords. In the motherese vs flat affect paradigm, the same actress was shown in both movies, reading from identical scripts, but in the contrasting movie, she used a flat affect, reading the script in a monotone voice. The movies were available for approximately 60 seconds, and toddlers used the direction of their gaze to control the amount of time each movie was played. Areas of interest (AOIs) were drawn on either side and consisted of each movie in its entirety (eg, motherese AOI or traffic AOI). To assess test-retest reliability, eye-tracking tests were repeated in a subset of toddlers 1 to 12 or more months following their initial eye-tracking session.

### Statistical Analyses

Primary analyses were conducted using R, version 4.0.2 (R Project for Statistical Computing), and SPSS, version 28 (IBM). Between-group differences were assessed using 1-way analyses of covariance with sex and age as covariates. The Tukey honestly significant difference test was used to perform post hoc, pairwise comparisons of group differences. Two-sided *P* < .05 indicated statistical significance. Ninety-five percent CIs of the mean differences between groups were calculated, and effect sizes are reported using η^2^ and Cohen *d*, as appropriate. Test-retest reliability was examined using intraclass correlations (ICCs; model 2, 1).^56^ Data were collected from February 2018 to April 2021 and were analyzed from April 2021 to March 2022.

#### Total Looking Time, Attention Toward Motherese Speech, and Number of Saccades

To assess whether overall attention differed between diagnostic groups, total looking time collapsed across AOIs was calculated for each paradigm. Time spent fixating within each AOI (eg, motherese or traffic) was divided by total looking time to determine a percent fixation value for each participant in each paradigm. The number of saccades per second was calculated for each participant as *n* – 1 fixations divided by the total looking time within motherese and nonmotherese AOIs.

#### Sensitivity, Specificity, Positive Predictive Value, Negative Predictive Value, and ROC Curve Analyses

For diagnostic classification accuracy analyses, toddlers were designated as having ASD or not having ASD (ie, ASD features, delay, typical sibling of an ASD proband, and TD) based on their most recent diagnostic evaluation. A receiver operating characteristic (ROC) curve was generated for each test and used to determine the optimal percent fixation and saccades per second associated with specificity of 96% or greater. A high specificity rate was chosen given the expense and anxiety associated with a false-positive result.^57^ To assess whether combining metrics enhanced classification accuracy, reduced error pruning was applied as the classifier within Waikato Environment for Knowledge Analysis (WEKA)^58,59^ using both percent fixation on motherese and saccades per second (eMethods in Supplement 1).

#### Association Between Motherese Fixation Levels, Social and Language Ability, and Age

Pearson correlations were used to examine motherese fixation and social and language associations (eMethods in Supplement 1). For interpretative simplicity, toddlers were also stratified into subgroups of high, medium, and low levels of fixation toward motherese, defined as follows: fixation of 30% or less, low motherese attention; fixation of 70% or more, high motherese attention; and the remaining toddlers, middle motherese attention. Differences in clinical profiles between each subgroup were examined.

## Results

Of 736 toddlers who participated in 1 or more motherese eye-tracking tests, 653 (88.72%) were included in the final analyses. The mean (SD) age was 26.45 (8.37) months; 173 (26.49%) were female, and 480 (73.51%) were male. A total of 15 (2.30%) were African American/Black; 8 (1.23%), American Indian/Alaska Native; 82 (12.56%), Asian; 5 (0.77%), Native Hawaiian/Pacific Islander; 365 (55.90%), White; and 67 (10.26%), more than 1 race; for 111 (17.00%), race was unknown, not reported, or other. A total of 225 (34.46%) were Hispanic or Latino and 369 (56.51%), not Hispanic or Latino; for 59 (9.03%), ethnicity was unknown or not reported. Of the 653 toddlers, 422 (64.62%) had ASD, 104 (15.93%) had non-ASD delays, and 127 (19.45%) had TD; 588 (90.05%) participated in the motherese vs traffic paradigm, 479 (73.35%) in the motherese vs techno paradigm, and 389 (59.57%) in the motherese vs flat affect paradigm. Toddlers with ASD represented a wide range of ability and disability in terms of adaptive behavior, cognitive and language ability, and autism symptom severity. Details regarding clinical score distributions are given in the Table and eFigure 4 in Supplement 1.

### Total Looking Time

There were no to minimal between-group differences in total looking time, depending on the paradigm. Details are given in the eResults in Supplement 1.

### Attention Toward Motherese Speech as Indexed by Percent Fixation

**Figure 1.**
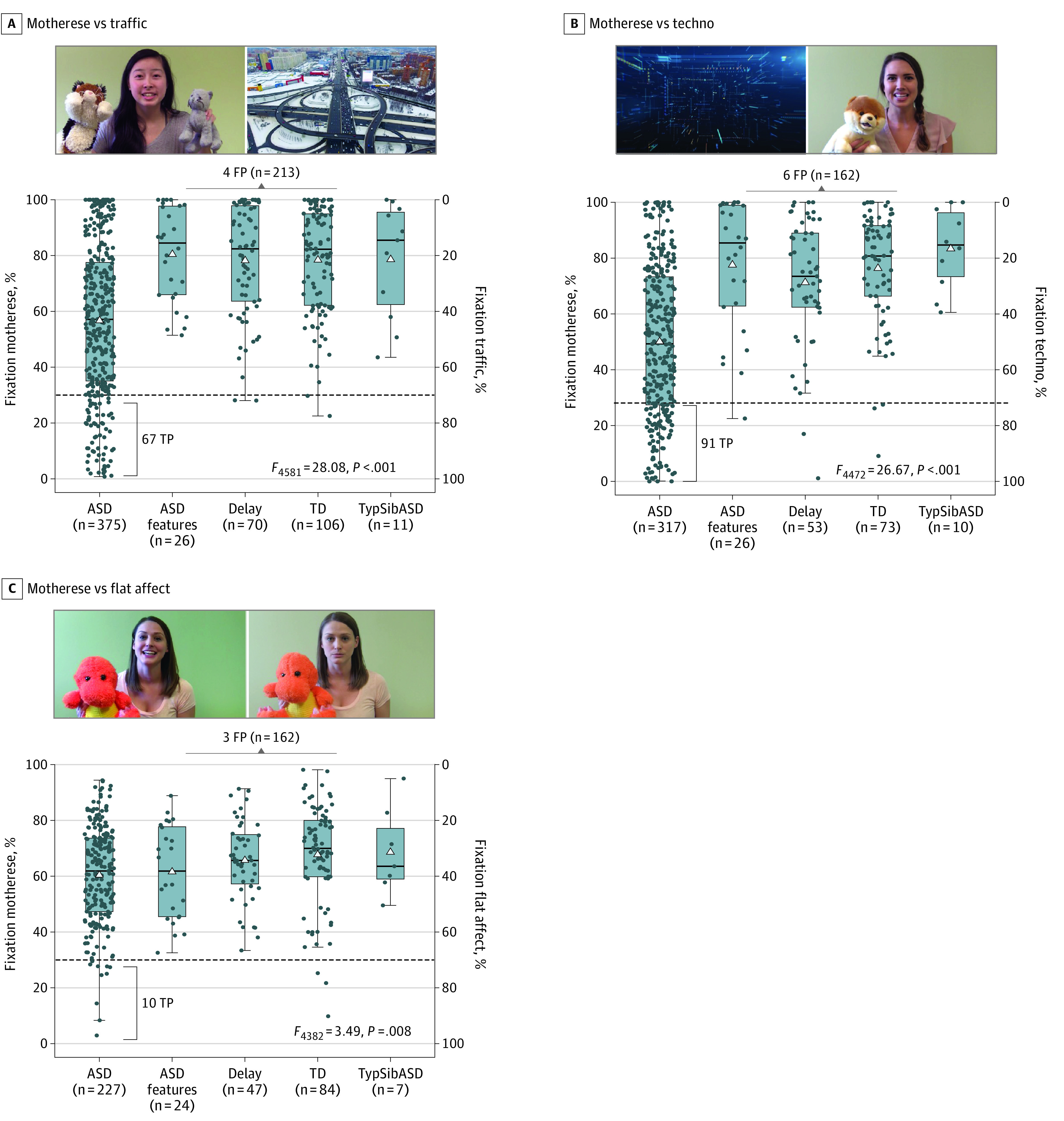
Motherese Eye-Tracking Test Performance Box plots show the raw data for each of the motherese eye-tracking tests across 5 major diagnostic groups. Middle line indicates median value; upper and lower bounds of the box, IQR; whiskers, range; dots, individual participants; and triangles, the mean. Because tests are all preferential looking paradigms, points along the y-axes sum to 100%; for example, within the motherese vs traffic paradigm, if a toddler fixated on motherese 90% of the time, he or she had a corresponding value of 10% fixation in traffic. ASD indicates autism spectrum disorder; FP, false positive; TD, typical development; TP, true positive; and TypSibASD, typical sibling of an ASD proband.

**Figure 2.**
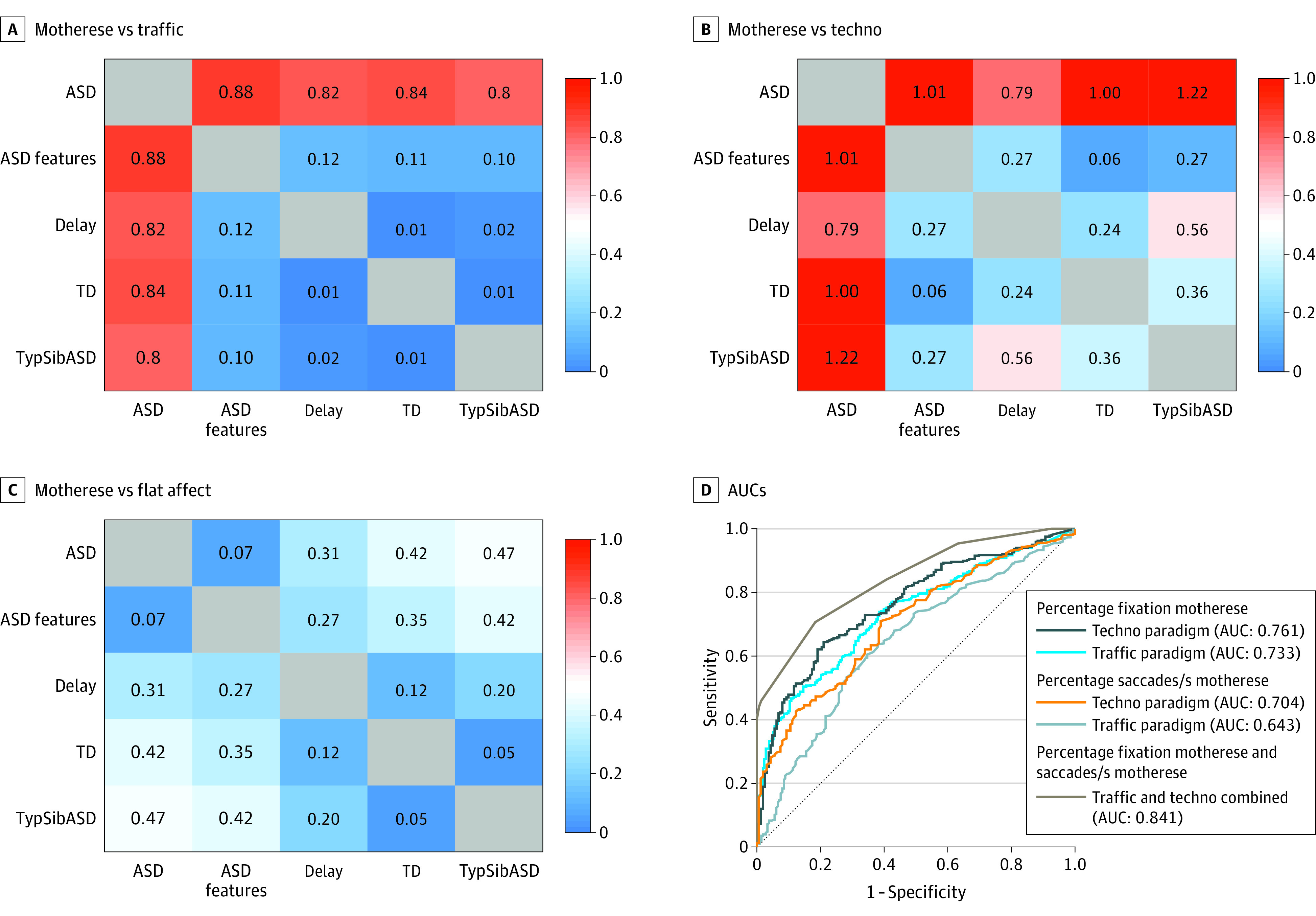
Effect Size and Area Under Receiver Operating Characteristic (ROC) Curve (AUC) A-C, Effect sizes indicate between-group differences. D, A reduced error pruning tree classifier using 4 eye-tracking metrics (percent fixation on motherese from the techno and traffic paradigms and saccades per second viewing motherese) was trained to classify toddlers who had participated in both the techno and the traffic paradigms (n = 430) as ASD (n = 283) or non-ASD (n = 147). ASD indicates autism spectrum disorder; TD, typical development; and TypSibASD, typical sibling of an ASD proband.

Unlike toddlers without ASD, who almost uniformly attended to motherese speech with a median level of 82.85% and 80.75% across the traffic and techno tests, respectively, among toddlers with ASD, there was a wide range spanning from 0% to 100%. Differences in percent fixation levels were significant across the traffic (*F*_4,581_ = 28.08; *P* < .001; η^2^ = 0.15) and techno (*F*_4,472_ = 26.67; *P* < .001; η^2^ = 0.18) paradigms, with toddlers with ASD fixating on the motherese videos significantly less than all other diagnostic groups (except motherese vs traffic among toddlers with ASD vs typical siblings of an ASD proband). For example, in the techno paradigm, the mean (SD) motherese fixation value for toddlers with TD was 76.36% (19.73%), whereas for toddlers with ASD, the mean was 50.08% (27.56%) (ASD vs TD mean [SD] difference, 26.28% [2.78%]; *P* < .001; Cohen *d*, 1.0). However, some toddlers with ASD had high levels of fixation on motherese at or near 100% values. Overall associated effect sizes were large for ASD vs other diagnostic groups, ranging from 0.79 to 1.22 across both paradigms. In contrast, there were only small between-group differences in the flat affect paradigm (*F*_4,382_ = 3.49; *P* = .008; η^2^ = 0.03) with associated small effect sizes (Figure 1 and Figure 2). Age was a significant covariate for both the traffic and flat affect paradigms (traffic, *F*_1,581_ = 22.66; *P* < .001; η^2^ = 0.03; flat affect, *F*_1,382_ = 11.27; *P* < .001; η^2^ = 0.03). Sex was a significant covariate only for the motherese vs traffic paradigm (*F*_1,581_ = 9.01; *P* = .003; η^2^ = 0.01). Pairwise comparisons of group differences and sex differences are given in the eResults, eTable 1, and eFigure 7 in Supplement 1.

### Number of Saccades

**Figure 3.**
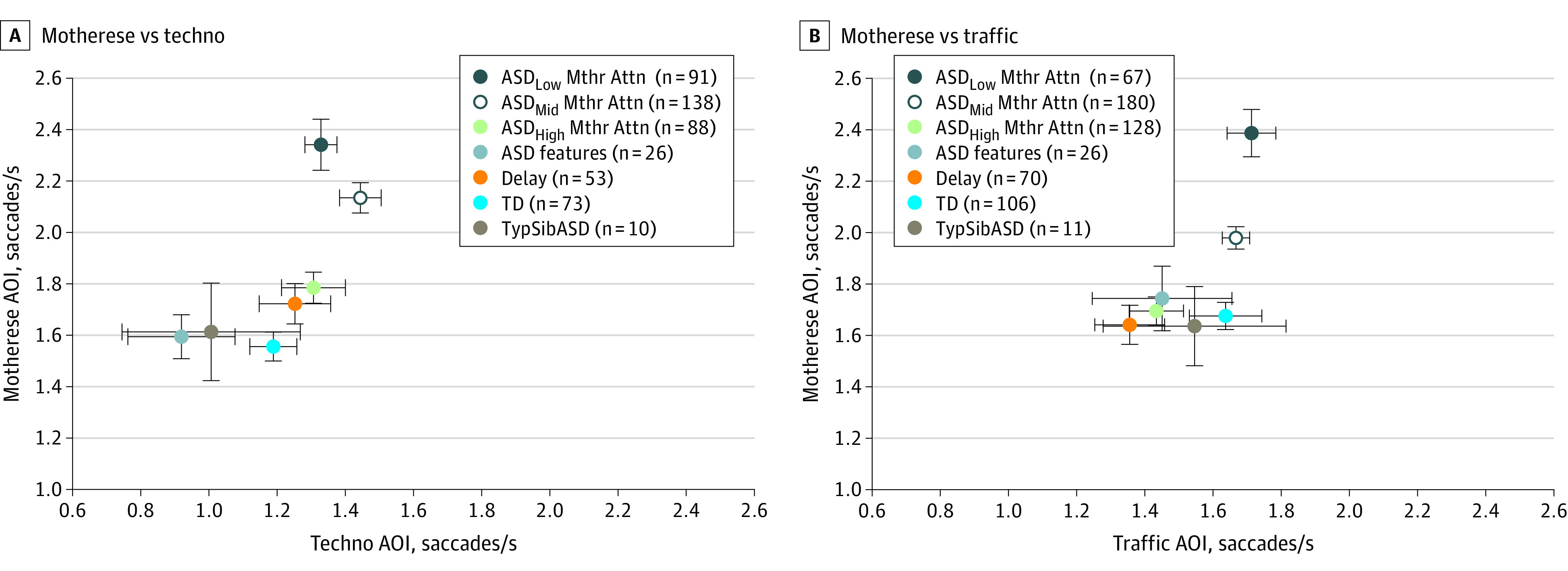
Saccade Profiles Stratified by Diagnostic Group Circles indicate the mean saccade rate and error bars indicate the standard error of the mean. Toddlers with autism spectrum disorder (ASD) were additionally stratified based on strength of preference for motherese. AOI indicates area of interest; Mthr Attn, motherese attention; TD, typical development; and TypSibASD, typical sibling of an ASD proband.

Similar to our eye-tracking tests of social visual attention,^32,33^ toddlers in the low motherese attention group showed a significantly increased number of saccades when viewing the social motherese AOIs (motherese vs techno paradigm: low motherese attention vs TD, 2.34 saccades vs 1.56 saccades; *F*_6,470_ = 3.17; *P* = .005; η^2^ = 0.04; motherese vs traffic paradigm: low motherese attention vs TD, 2.39 saccades vs 1.68 saccades; *F*_6,579_ = 14.88; *P* < .001; η^2^ = 0.13). Conversely, across diagnostic groups, saccade rates were largely comparable when attending to nonsocial AOIs (Figure 3). Correlations across paradigms are given in the eResults and eFigure 8 in Supplement 1.

### Sensitivity, Specificity, Positive Predictive Value, Negative Predictive Value, and ROC Curve Analyses

Using a value of 30% fixation or less on motherese as the cutoff value resulted in sensitivity of 18% (95% CI, 14%-22%), specificity of 98% (95% CI, 95%-99%), positive predictive value (PPV) of 94% (95% CI, 86%-98%), and negative predictive value (NPV) of 40% (95% CI, 36%-45%) and an associated area under the ROC curve (AUC) of 0.733 (95% CI, 0.693-0.773) for the traffic paradigm and 29% (95% CI, 24%-34%) sensitivity, 96% (95% CI, 92%-98%) specificity, 94% (95% CI, 86%-98%) PPV, and 41% (95% CI, 36%-46%) NPV and an associated AUC of 0.761 (95% CI, 0.717-0.804) for the techno paradigm. In contrast, values were low for the flat affect paradigm, which had an AUC of 0.597 (95% CI, 0.541-650). Combining saccades per second with percent fixation metrics across the traffic and techno paradigms increased the AUC to 0.841 (95% CI, 0.805-0.877). Diagnostic accuracy cross-tabulation tables and 95% CIs are provided in eTables 2 and 3 in Supplement 1. Given the lack of diagnostic classification accuracy and small effect sizes, the flat affect paradigm was not considered in further analyses.

### Association Between Motherese Fixation Levels, Social and Language Ability, and Age

**Figure 4.**
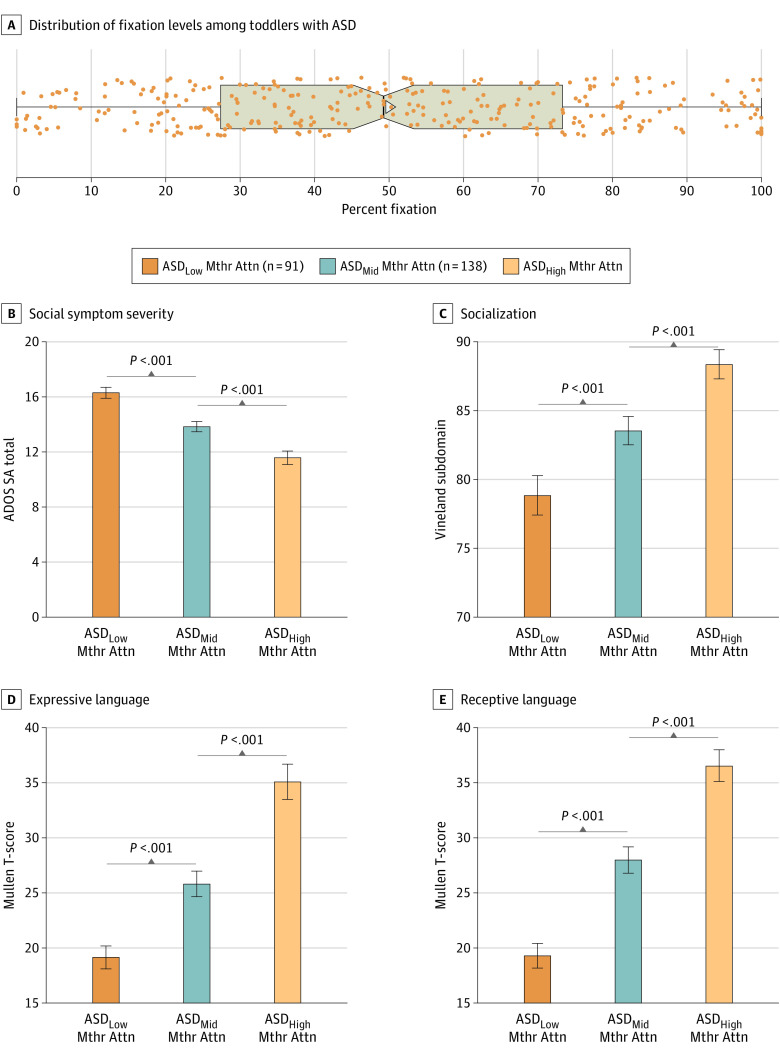
Differences in Clinical Phenotype Among Toddlers With Autism Spectrum Disorder (ASD) With Low, Middle, and High Attention to Motherese Speech During the Techno Paradigm A, Dots indicate percent fixation values on motherese for individual toddlers; whiskers, the range; and triangle, the mean. B-E, Differences in standardized test scores between subgroups. Results were similar for the traffic paradigm (eResults and eFigure 9 in Supplement 1). ADOS indicates Autism Diagnostic Observation Schedule; Mthr Attn, motherese attention; and SA, social affect.

Collapsed across all diagnostic groups as well as within the ASD group alone, percentage of time fixating on motherese speech during the techno paradigm was significantly correlated with receptive and expressive language ability, social affect scores, and the overall socialization domain score on the Vineland scales (Pearson *r* range for toddlers with ASD: 0.30-0.48; *P* < .001). When toddlers with ASD were stratified as having low, middle, or high levels of attention toward motherese speech, significant differences in social and language abilities between the subtypes were observed. For example, within the social domain, the mean (SD) Vineland score was 9.55 (1.78) points lower (Cohen *d*, 0.79; *P* < .001) and the mean (SD) social affect total score was 4.73 (0.63) points higher (indicating greater symptom severity) in the low motherese attention group than in the high motherese attention group (Cohen *d*, −1.12; *P* < .001). Within the expressive and receptive language domains, *t* scores were a mean (SD) of 16.03 (1.90) and 17.45 (1.84) points lower, respectively (expressive: Cohen *d*, 1.26 [*P* < .001]; receptive: Cohen *d*, 1.42 [*P* < .001]) (Figure 4). Similar results were obtained for the traffic paradigm (eFigure 9 in Supplement 1). Overall, there were inconsistent associations between fixation on motherese and age across diagnostic groups and paradigms (eResults, eTable 4, and eFigures 9 and 10 in Supplement 1).

### Test-Retest Reliability

Percent fixation levels on motherese up to 1 year following initial testing were significantly correlated in the traffic paradigm, with ICC values ranging from 0.38 to 0.77. Fixation values associated with intervals greater than 1 year were significantly correlated only in the techno paradigm (ICC, 0.48; *P* = .001) (eResults and eTable 5 in Supplement 1).

## Discussion

The animated speech style known as motherese can be found across cultures and languages,^60^ is evident between deaf mothers and their deaf children,^61,62^ and has even been noted to occur while fetuses are still in the womb.^63^ Likely genetic, motherese is more than a speech style; it is a tool for reciprocal engagement that enhances social and language development, with infants often matching the fundamental frequencies and voice pitch of their mothers.^64^ Using gaze-contingent eye tracking that allowed toddlers to control the images and sounds they heard, we found that similar to toddlers who are typically developing or non–ASD delayed, the majority of toddlers with ASD attended to motherese speech at high levels. In contrast, approximately 23% of toddlers with ASD showed low levels of attention toward motherese speech, with some as low as 1% to 2%. This subgroup instead preferred to listen to noises such as the sound of horns beeping and car engines during traffic. When fixation levels on motherese were at or below 30%, the PPV and specificity across the motherese vs traffic and motherese vs techno paradigms were high and ranged between 94% and 98%, depending on the test. Given that the tests are engaging for toddlers, result in objective quantitation of social and language attention, and require less than 5 minutes to complete, they may be useful as a screening or diagnostic tool to identify toddlers in most need of immediate attention.

Our conservative selection of a cutoff value of 30% fixation or less on motherese was deliberate to tune the tests to have high specificity and reduce false-positive results, which are unnecessarily stressful for parents.^57^ There is often a tradeoff, however, between specificity and sensitivity, and this deliberate tuning lowered sensitivity. The low sensitivity of the motherese-attention biomarkers and other ASD biomarkers^31^ likely reflects the true clinical and biological heterogeneity inherent in ASD.^65,66,67^ In terms of genetic findings, most single-gene sequence variations, such as in the Shank gene family, are found in approximately 1% of children with ASD.^68^

There was also an association between level of attention to motherese speech and social and language abilities, with children showing the lowest levels of interest in motherese speech also having the lowest social and language abilities. This is not surprising given the association of experience with brain development.^25,69,70,71,72^ Stratifying toddlers with ASD in the current study into low, middle, and high levels of attention to motherese speech also highlighted the reverse phenomenon: toddlers in the high–motherese attention group had receptive and expressive language standard scores 17.45 and 16.03 points, respectively, above those of toddlers with ASD in the low–motherese attention group as well as better social affect scores. Given that there are critical periods for language development that extend across the first years of life,^73^ it may be even more challenging for children with ASD in the low–motherese attention group to catch up once this critical period has passed. At least 1 previous eye-tracking study^74^ showed that toddlers with ASD who paid the least attention to social images at ages 1 to 3 years had greater symptom severity 5 to 9 years later.

Our data also suggest that the subgroup of the population with ASD and low attention to motherese may be more homogeneous at some intermediate psychobiological level, thereby potentially justifying the use of this test to stratify the population with autism in future studies of social attention and its correlates. A recent study^27^ revealed that toddlers with ASD who showed low attention levels to motherese speech as a group had lower levels of neural functional activity in classic speech-processing areas, such as the superior temporal gyrus, compared with other toddlers with ASD who paid greater attention to motherese speech.

Our findings also revealed a dissociation between saccade rates depending on what toddlers were looking at but only for the ASD subgroup that showed the lowest levels of attention to motherese. These toddlers showed a greater number of saccades during the brief periods that they viewed the motherese videos, possibly reflecting greater anxiety or challenges related to speech processing.

Interestingly, the motherese vs flat affect paradigm was not effective as a diagnostic classifier, showing only minimal between-group ASD vs TD differences. Given that null results are rarely reported,^75^ we believed this was important to underscore. One reason may be related to the nature of the flat affect video that contained an actress speaking in an unnatural computer-like voice that caused several toddlers to smirk and may have artificially increased attention to this video. Indeed, total looking time in the flat affect AOI was higher than in the nonmotherese contrast videos used in the other paradigms.

### Strengths and Limitations

This study has several strengths. These include comprehensive diagnostic and psychometric evaluations conducted by licensed clinical psychologists blind to eye-tracking scores and the inclusion of multiple contrast groups including toddlers with non-ASD delays, an essential component for determining future clinical utility.

This study also has limitations. Given that the sample contained a combination of toddlers who first failed a parent-report screen at well-infant checkups and general community referrals, a key limitation relates to the unknown generalizability of the findings as a universal screening approach.

## Conclusions

Overall, attention to motherese speech is a fundamental property of early development that can be rapidly measured using eye tracking. In this diagnostic study, a cutoff level of 30% fixation or less on motherese speech was accurate at identifying toddlers who were diagnostically classified as having ASD. Identifying toddlers who show unusually low levels of attention to motherese speech is potentially beneficial not only for early ASD screening, diagnosis, and prognosis but also for possible therapeutic targets and may be a key pathway toward precision medicine.

## Notes

Supplement 1.
eMethods.
**eFigure 1**. Participation Flowchart **eFigure 2.** Summary of Reasons for Exclusion **eFigure 3.** Test Overlap and Intersection Plot **eFigure 4.** Histograms Illustrating Distribution of Clinical Scores Across Diagnostic Groups **eFigure 5.** Comparison of Motherese Fixation Levels Across Eye-Tracking Systems **eFigure 6.** Preexperiment Dog-Cat Training Task
eResults.
**eTable 1.** Pairwise Comparisons of Diagnostic Group Differences Across Motherese Paradigms **eTable 2.** Cross-tabulation Tables **eTable 3.** Metrics of Diagnostic Accuracy with 95% CIs **eFigure 7.** Motherese Fixations Levels Stratified by Sex **eFigure 8.** Relationships Between Motherese Fixation Levels Across Motherese Paradigms **eTable 4.** Correlations Between Motherese Fixation Levels and Social and Language Ability in the Techno and Traffic Paradigms **eFigure 9.** Differences in Clinical Phenotype Between Toddlers with Low, Middle, and High Attention to Motherese Speech During the Traffic Paradigm **eFigure 10.** Age-related Changes in Motherese Fixation Levels **eTable 5.** Test-Retest Reliability
eReferences.

Supplement 2.
Data Sharing Statement
