# Supplementary material for: Level of Attention to Motherese Speech as an Early Marker of Autism Spectrum Disorder
Source: JAMA Netw Open. 2023 Feb 8;6(2):e2255125. doi: 10.1001/jamanetworkopen.2022.55125 (PMC9909502; doi:10.1001/jamanetworkopen.2022.55125)
Supplement: Supplement 1. — eMethods. eFigure 1. Participation Flowchart eFigure 2. Summary of Reasons for Exclusion eFigure 3. Test Overlap and Intersection Plot eFigure 4. Histograms Illustrating Distribution of Clinical Scores Across Diagnostic Groups eFigure 5. Comparison of Motherese Fixation Levels Across Eye-Tracking Systems eFigure 6. Preexperiment Dog-Cat Training Task eResults. eTable 1. Pairwise Comparisons of Diagnostic Group Differences Across Motherese Paradigms eTable 2. Cross-tabulation Tables eTable 3. Metrics of Diagnostic Accuracy with 95% CIs eFigure 7. Motherese Fixations Levels Stratified by Sex eFigure 8. Relationships Between Motherese Fixation Levels Across Motherese Paradigms eTable 4. Correlations Between Motherese Fixation Levels and Social and Language Ability in the Techno and Traffic Paradigms eFigure 9. Differences in Clinical Phenotype Between Toddlers with Low, Middle, and High Attention to Motherese Speech During the Traffic Paradigm eFigure 10. Age-related Changes in Motherese Fixation Levels eTable 5. Test-Retest Reliability eReferences. [file jamanetwopen-e2255125-s001.pdf]

Supplementary Online Content

Pierce K, Wen TH, Zahiri J, et al. Level of attention to motherese speech as an early marker of autism spectrum disorder. *JAMA Netw Open*. 2023;6(2):e2255125. doi:10.1001/jamanetworkopen.2022.55125

eMethods.
eFigure 1. Participation Flowchart
eFigure 2. Summary of Reasons for Exclusion
eFigure 3. Test Overlap and Intersection Plot
eFigure 4. Histograms Illustrating Distribution of Clinical Scores Across Diagnostic Groups
eFigure 5. Comparison of Motherese Fixation Levels Across Eye-Tracking Systems
eFigure 6. Preexperiment Dog-Cat Training Task
eResults.
eTable 1. Pairwise Comparisons of Diagnostic Group Differences Across Motherese Paradigms
eTable 2. Cross-tabulation Tables
eTable 3. Metrics of Diagnostic Accuracy with 95% CIs
eFigure 7. Motherese Fixations Levels Stratified by Sex
eFigure 8. Relationships Between Motherese Fixation Levels Across Motherese Paradigms
eTable 4. Correlations Between Motherese Fixation Levels and Social and Language Ability in the Techno and Traffic Paradigms
eFigure 9. Differences in Clinical Phenotype Between Toddlers with Low, Middle, and High Attention to Motherese Speech During the Traffic Paradigm
eFigure 10. Age-related Changes in Motherese Fixation Levels
eTable 5. Test-Retest Reliability
eReferences.

This supplementary material has been provided by the authors to give readers additional information about their work.

© 2023 Pierce K et al. *JAMA Network Open*.

eMETHODS

Study Design

The current study is a prospective diagnostic study designed to evaluate the diagnostic accuracy of ‘motherese’ eye tracking tests. Subjects were enrolled in the study from 2/5/2018 to 4/29/2021 at the University of California, San Diego Autism Center. General data collection methods and procedures were based on our previous eye tracking work[1-4], and was planned before the index tests (i.e., the *Techno and Traffic,* motherese eye tracking tests) and reference standard (i.e., the Autism Diagnostic Observation Scale (ADOS), DSM-5 criteria, and clinical judgement, see below) for the current study were performed. Subjects were enrolled consecutively.

Subject Recruitment

The *Get SET Early* ASD detection program[5] focuses on a three-step process where S=Screen, E=Evaluate, and T=Treat, linking the critical elements of screening, evaluation, and treatment in a rapid fashion. The overall goal of the model is to detect all cases of ASD within a pediatric office by the second birthday. To support the first step of screening, a local pediatrician network of 203 pediatricians at 37 offices was established and trained to administer a parent report broadband screening tool, the CSBS IT Checklist[6], to all toddlers at 12, 18, and 24 month well baby visits. Pediatricians were provided training on the scoring, interpretation of screening, and identification of early developmental delays. Pediatrician office staff were trained to refer any children with developmental concerns based on a failed CSBS or pediatrician concern to our Center for further evaluation. As part of the model, a state-of-the-art evaluation center with autism expert evaluators was established to ensure rapid and accurate assessment of delays. All clinicians were PhD level licensed psychologists with expertise with toddlers with autism. Immediately following the evaluation, test results were reviewed with the parents and referrals were made for appropriate community treatments the same day. Overall, this three-step model allows for a thorough yet rapid process that detects delays at very young ages and engages families in early intervention as soon as a delay is detected.

Community Self-Referred Sample. Due to our Center’s high profile within the community, ~25% of our sample is based on community referrals outside of the *Get SET Early* Model. Parents of these toddlers sought out a developmental evaluation independent of their pediatrician due to their own concerns about their child’s development. In some cases, parents were referred to our Center by professionals in the community (e.g., a speech therapist). Once a connection is established at our Center, self-referred families proceed through the same process of evaluation and referral for treatment as toddlers detected by the *Get SET Early* model.

Given that the majority of the sample was ascertained via universal screening wherein all toddlers were screened at well-baby visits, our cohort is likely a valid representation of the general population within San Diego County. Moreover, our unique subject ascertainment approach has resulted in one of the youngest and largest general population samples in the literature to date.

Eligibility Criteria. With the exception of toddlers who were blind, deaf, or had known vision abnormalities, all toddlers were eligible for participation between the ages of 12-48 months.

Intended Sample Size and How Determined. Intended sample size was determined informally by leveraging several considerations. Given the large ASD vs TD effect sizes for the *Techno* and *Traffic* eye tracking paradigms, power analyses conducted at the outset of the study revealed that only very small samples sizes (~10-16 per group) would be required to reveal significant between-group effects. Considerably larger sample sizes, however, would be required for diagnostic classification accuracy analyses, and calculations of minimum sample sizes required depend on pre-specified values of power, p-value, effect size as well as disease prevalence rates. In the current study the majority of subjects were referred by pediatricians following screen failure in a pediatric office, resulting in a disease prevalence in the overall sample of ~50%. According to sample size tables provided by[7], this would result in a required sample size of ~155 ASD subjects and an overall sample size of 310. Our prior eye tracking studies however, revealed considerable heterogeneity in eye tracking data in ASD, with approximately 20% of toddlers with ASD exhibiting extreme eye tracking values[4,8], that would support revealing unique subtypes. Thus in the current study an informal goal of obtaining at least 200 toddlers with ASD and 200 toddlers with non-ASD delays or were typically developing was set. This sample size is beyond the minimum stated by[7] and would allow us to reveal subgroups within the ASD group that exhibited extremely eye tracking profiles, even if this subgroup made up only 10-20% of the sample.

Diagnostic Criteria

All diagnoses were based on testing performed by licensed clinical psychologists, blind to eye tracking scores. A toddler was designated in each of the following diagnostic categories based on their most recent evaluation visit using the following criteria: *Autism Spectrum Disorder (ASD)* - scored within the range of concern on the Autism Diagnostic Observation Schedule-2 (ADOS)[9,10] using pre-defined cut off scores defined by the test developer[9,10] and was considered ASD based on DSM 5 criteria[11] and clinical judgment. Combining results from the ADOS, DSM-5 and clinical judgement is considered the best available method for establishing the presence of autism and is used as the reference standard in the current study. *ASD Features* - showed signs of autism and may have an elevated ADOS score but did not meet full criteria for ASD. *Developmental Delay* included toddlers that displayed either a language delay defined as > 1 standard deviation below expected values on either or both the receptive or expressive subtests on the

© 2023 Pierce K et al. *JAMA Network Open*.

Mullen Scales of Early Learning; global developmental delay defined as > 1 standard deviation below expected values on two or more areas of the Mullen with at least one of those areas outside of the verbal scales; or toddlers with other delays including motor delay and social emotional delay. Toddlers were determined to be *Typically Developing, (TD)*, if they fell within the normal range on all clinical assessments and *TypSibASD* if they also had sibling with ASD.

Time Interval Between Eye Tracking Test and Final Diagnosis

Although research has shown that the diagnosis of ASD is relatively stable starting at 14 months[12], whenever possible, toddlers that participated in eye tracking and received their diagnosis ≤ 30 months were invited for longitudinal follow up testing. In the current study 68% of toddlers received eye tracking and 'final' diagnosis on the same day (mean age at eye tracking and diagnosis, 28.91 months). Thus, the time interval between eye tracking and final diagnosis was zero. For the remaining toddlers, final diagnosis occurred 13.8 months following initial eye tracking (mean age at eye tracking 23 months, mean age of final diagnosis 36.8 months). In the current study, the most recent diagnosis was always used in classification analyses.

STARD Criteria

Standards for Reporting Diagnostic accuracy studies (STARD)[13] were initially developed to contribute to the completeness and transparency of reporting of diagnostic accuracy studies. The current study followed the 30 items outlined in the 2015 guidelines[13].

Mullen estimated T score

The lowest minimum subscale T score based on the Mullen scoring manual is 20. However, some toddlers with ASD and DD in our study performed at levels that were below 20, particularly in the receptive and expressive language subdomains. In these instances, we elected to generate a T score for each toddler that was an approximate reflection of ability based on their raw score and chronological age, rather than artificially assigning all such toddlers a T score of 20. Among toddlers who received the *Motherese vs. Techno* paradigm, Expressive Language T scores were estimated for 22.54% (i.e., 108 toddlers) of the sample, and Receptive Language T scores were estimated for 12.53% (i.e., 60 toddlers) of the sample. Among toddlers who received the *Motherese vs. Traffic* paradigm, Expressive Language T scores were estimated for 21.43% (i.e., 126 toddlers) of the sample, and Receptive Language T scores were estimated for 10.37% (i.e., 61 toddlers) of the sample. For these toddlers, T-scores were estimated using the raw scores obtained with the table for the child's chronological age, as follows. The estimated T score is

© 2023 Pierce K et al. *JAMA Network Open*.

calculated by examining the variation in raw scores for the lowest T scores available for the child's age and applying that variation to estimate a lower T score. For example, if the lowest raw score available for the child's age is 14, but the child actually has a raw score of 12, two steps would be counted down and an estimated score would be calculated based on the amount of difference between T scores for each raw score above the cut off. So, if the raw score of 14 corresponded to a T of 20, and there was a 2 point difference between each T score above 20, then the estimated T score would be 16 (2 steps times 2 point difference = 4 and thus the estimated T score is 20-4 =16).

Subject Exclusion Criteria and Examination of Clinical Differences Between Included and Excluded

Overall, only 11% of toddlers that attempted eye tracking were unable to complete at least 1 test. However, rates of successful data collection varied across paradigm. For *Motherese vs. Traffic*, 696 toddlers initially attempted eye tracking, and 108 (75 ASD and 33 NonASD) were excluded from analyses. For *Motherese vs. Techno*, 566 toddlers initially attempted eye-tracking, and 87 (61 ASD and 26 NonASD) were excluded from analyses. For *Motherese vs. Flat Affect*, 513 toddlers initially attempted eye-tracking, and 124 (94 ASD and 30 NonASD) were excluded from analyses. Reasons for exclusion are detailed in **eFigure 2**. Among ASD toddlers who completed the *Motherese vs. Traffic* paradigm, symptom severity as indexed by the ADOS total score was not different between included and excluded toddlers ($t(106.75) = 0.82$, $p=0.41$, *ns.*). Among ASD toddlers who completed the *Motherese vs. Techno* or *Motherese vs. Flat Affect* paradigm, symptom severity was significantly different (*Motherese vs. Techno*: $t(93.09) = 2.64$, $p<0.01$; *Motherese vs. Flat Affect:* $t(179.31) = 5.06$, $p<0.0001$).

Eye Tracking Testing Overlap

A majority of toddlers participated in more than one Motherese eye tracking test. For example, as illustrated in the intersection plot in **eFigure 3**, 430 toddlers participated in both the *Motherese vs Traffic* and *Motherese vs Techno* paradigms.

Tobii T120 and Tobii Pro Spectrum Eye Trackers Participation

Eye tracking data was collected for this study from February 2018-April 2021. Data was collected using a Tobii T120 at a sampling rate of 60 Hz for 365 toddlers who participated from February 2019 to July 2019 and using a Tobii Pro Spectrum eye tracker at a sampling rate of 600 Hz for 288 toddlers who participated from July 2019 to April 2021. Given differences in the number of subjects per diagnostic category, a random sample of 50 ASD and 50 NonASD subjects recorded on each eye tracker were obtained to compare Motherese fixation levels. For the *Motherese vs. Flat Affect* paradigm, 40 NonASD subjects were randomly sampled from each eye tracker as there were only 40 NonASD subjects recorded using Tobii

© 2023 Pierce K et al. *JAMA Network Open*.

Pro Spectrum. As see in ***eFigure 5***, across all three Motherese paradigms, fixations levels were comparable across machines for both ASD and NonASD toddlers (*Motherese vs. Traffic:* ASD t(97.22)= -1.92, p=0.06, *ns*., NonASD t(92.93)= -1.90, p=0.06, *ns*.; Motherese vs Techno ASD: t(97.62)= -0.80, p=0.43, *ns*., NonASD t(97.99)= -0.80, p=0.42, *ns*.; *Motherese vs. Flat:* ASD t(97.01)=-1.80 p=0.08, *ns*.; NonASD t(77.82)=-0.03, p=0.97, *ns*.). As such, data from both machines was collapsed and analyzed together for subsequent analyses.

Eye Tracking Procedure and Calibration

Procedure. Prior to entering the eye tracking room, parents were read a series of standardized instructions by an eye tracking technician describing the eye tracking procedure and were asked not to direct their child's attention, or to look directly at the images but to instead look at a circle placed 3 feet above the eye tracker. Toddlers were seated on their parent's lap 60 cm in front of the eye-tracking monitor. Eye tracking technicians were blind to diagnostic status at the time of testing.

Calibration and Fixation Filter. A five-point calibration was performed using animated cartoon ducks with sounds, and data was only used if calibration results, determined via graphical output (T120) or reported using accuracy and precision values (Pro Spectrum 600) fell within manufacturer-reported parameters (accuracy, .5 degrees[14]). Tobii software was used to record both fixation duration and number of saccades during each paradigm. Fixations were determined by a 35-pixel radius filter using Tobii Software (www.Tobiipro.com).

Central Fixation. To standardize toddlers' starting attentional focus, each paradigm was preceded by a flashing smiley face programmed such that central fixation was required to start the experiment.

Pre-Experiment Dog Cat Gaze Contingent Training

To introduce toddlers to the concept of gaze contingency, toddlers were shown images of a puppy and a kitten placed side-by-side wherein fixation on the kitten triggered a movie depicting the kitten purring, while fixation on the puppy triggered a movie of the puppy gently barking. Training continued until toddlers made at least 3 alternating fixations between both sides of the movie (i.e., showed volitional control in triggering specific movie files). If a toddler did not spontaneously alter their gaze between the movies within 20 seconds, the experimenter pointed to one side and said "Look at the Dog/Cat!". Out of the 653 toddlers in the study, with only 1 exception, all made at least 3 gaze alterations, indicating some awareness of the gaze contingent nature of the task. Interestingly, many toddlers showed a clear preference for either the cat or the dog. **See eFigure 6.**

Statistical Analyses

© 2023 Pierce K et al. *JAMA Network Open*.

Primary analyses were conducted using R and SPSS. Between group differences were determined using one-way ANCOVAs with sex and age as covariates. Tukey's HSD was used to perform post-hoc, pairwise comparison of group differences. Ninety five percent confidence intervals of the mean difference between groups were calculated, and effect sizes reported using Cohen's d. Test-retest reliability was examined using intraclass correlations (ICCs; model 2, 1)[15].

Relationship Between Motherese Fixation Levels, Social and Language Ability and Age. Pearson's correlations were used to examine relationships between percent fixation on motherese and social ability as indexed by the Social Affect score on the ADOS or the Socialization subdomain score on the Vineland, and language as indexed by the receptive and expressive subscales of the Mullen as well as with age.

Classification Accuracy Using Reduced Error Pruning (REP)

A reduced error pruning (REP) decision tree was applied using both percent fixation on motherese and saccades per second to classify subjects as ASD or nonASD. REP tree uses information gain as the splitting criterion. Pruning was done using reduced-error pruning with back fitting. We used REP tree implementation in Waikato Environment for Knowledge Analysis (WEKA; https://doi.org/10.1145/1656274.16562780). To avoid overfitting, the depth of the tree was limited to four. We applied this classifier to the subset of toddlers who participated in both the *Techno* and *Traffic* motherese tests (N=430; ASD 283, nonASD 147). Therefore, there were no missing values.

© 2023 Pierce K et al. *JAMA Network Open*.

Participation Flowchart

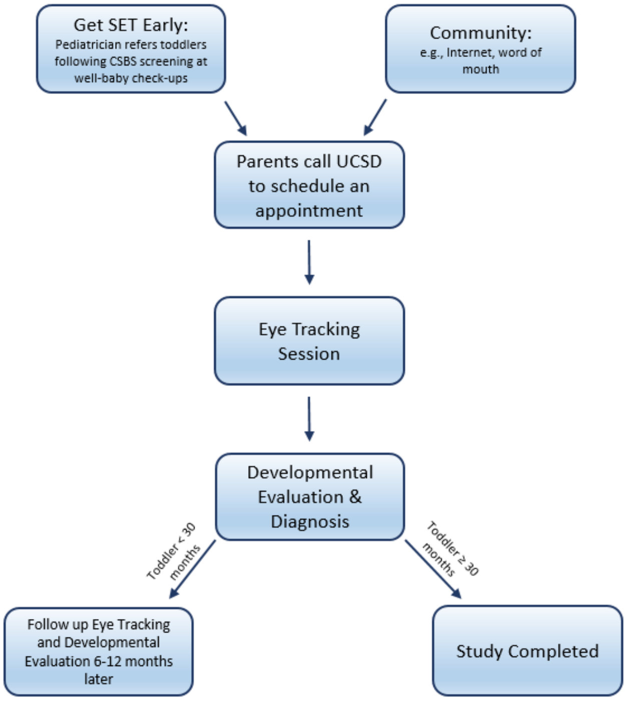

eFigure 1. Participation Flowchart. SET=Screen Evaluate Treat

© 2023 Pierce K et al. *JAMA Network Open*.

eFigure 2. Summary of Reasons for Exclusion. Bar graphs showing the number of toddlers excluded for each Motherese test due to a variety of reasons including calibration issues, parent interference, or poor child behavior (e.g., restlessness, wiggling, tantrums, etc.). Toddlers excluded due to vision abnormalities was based on parent report and includes strabismus, astigmatism, and myopia or hyperopia that requires wearing corrective glasses.

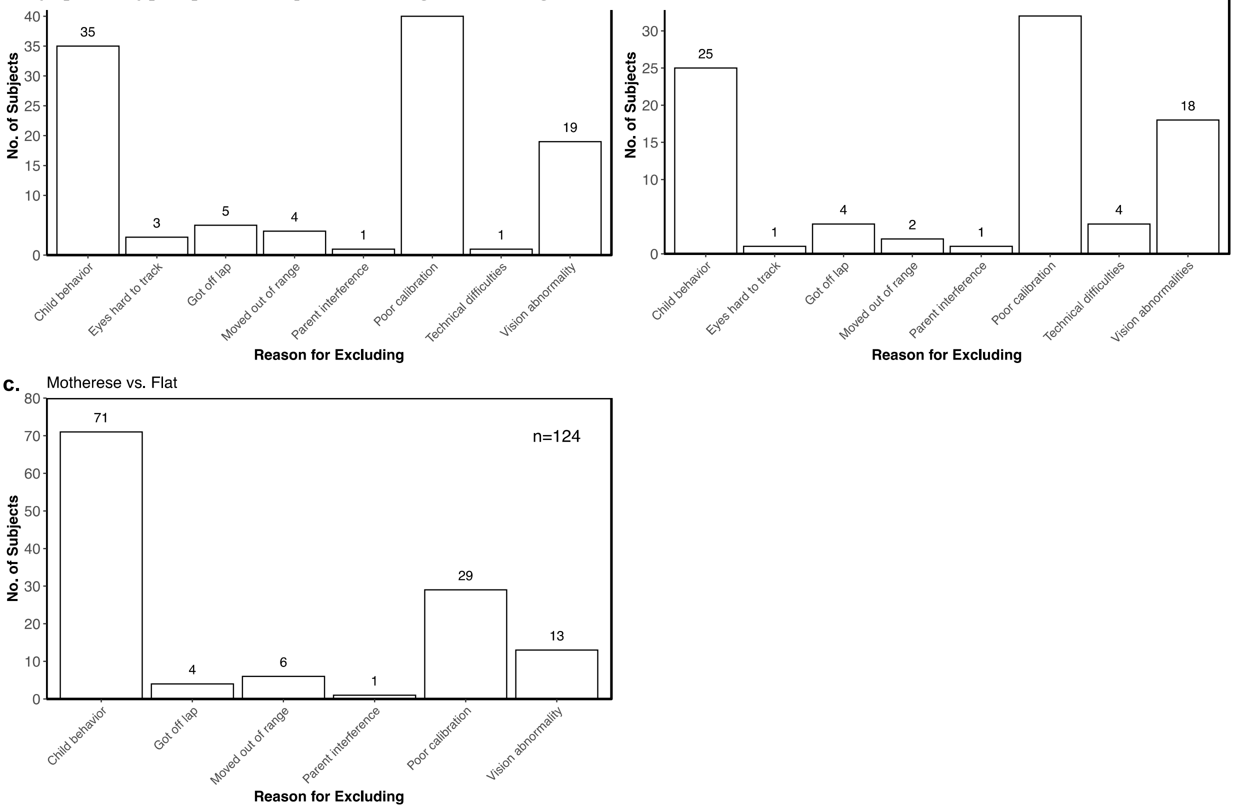

© 2023 Pierce K et al. *JAMA Network Open*.

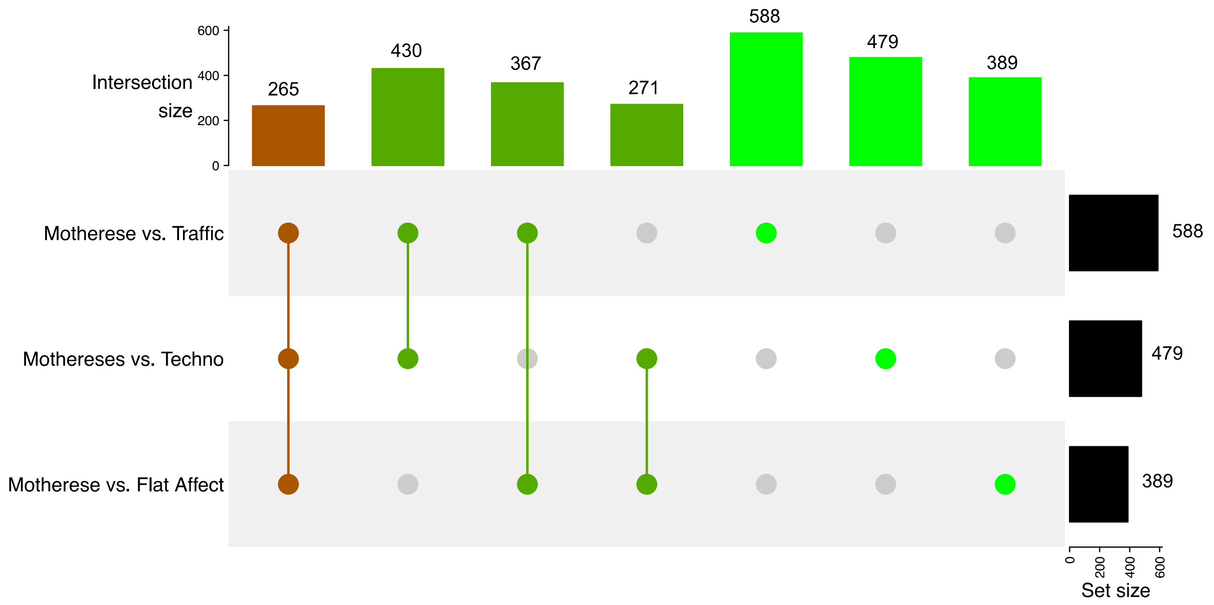

eFigure 3. Test Overlap/Intersection Plot. Graphical illustration of the number of toddlers that participated in each eye tracking test and associated overlap. The small numbers on top indicate the number of toddlers that received a particular 'intersection' of tests. For example, 265 toddlers received all 3 Motherese tests. The numbers at the end of each row indicate the total number of eye tracking tests available for each test. For example, 588 toddlers participated in the Motherese vs. Traffic paradigm.

© 2023 Pierce K et al. *JAMA Network Open*.

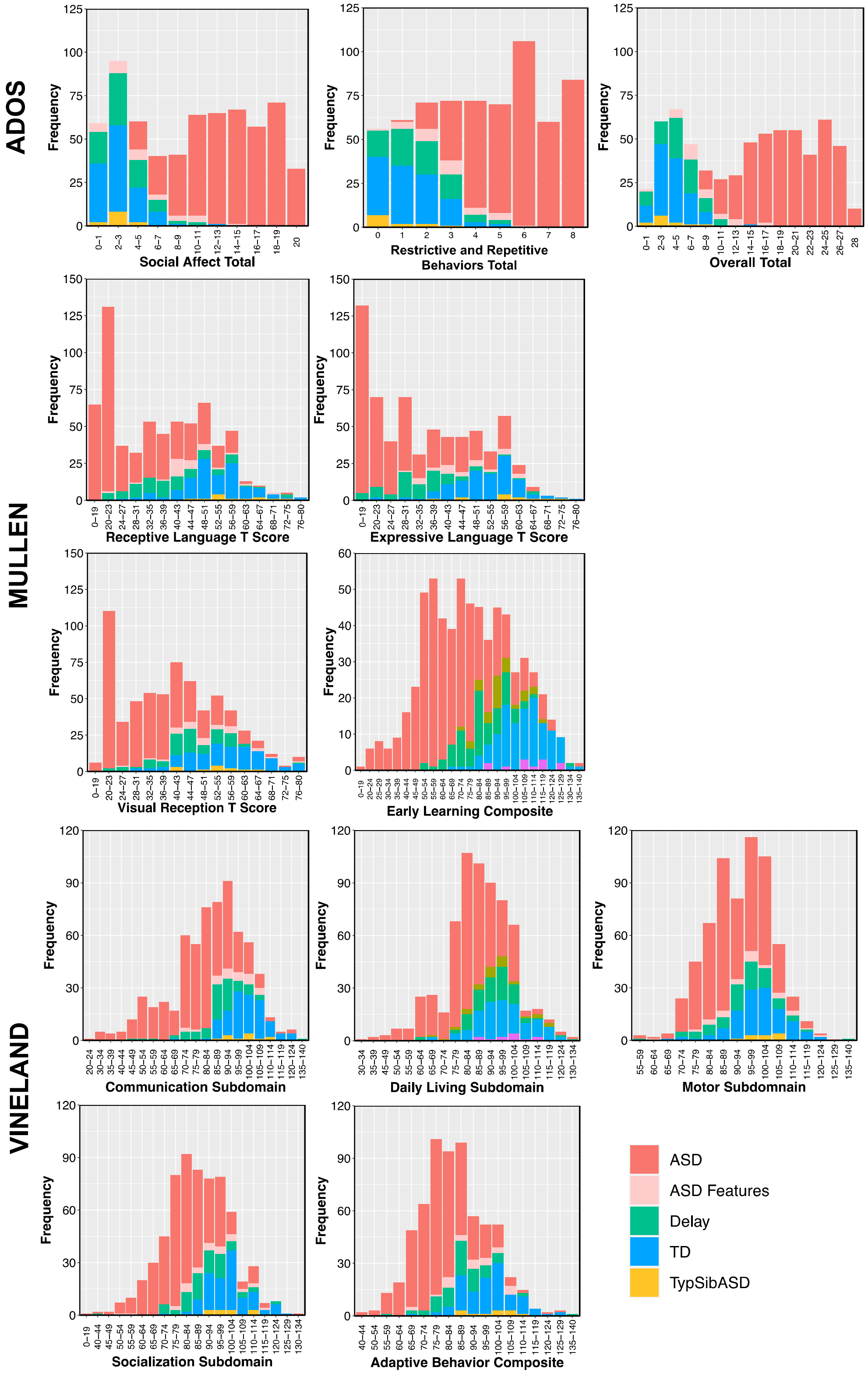

eFigure 4. Histograms Illustrating Distribution of Clinical Scores Across the ADOS, Mullen and Vineland, Stratified by Diagnostic Group. ASD=Autism Spectrum Disorder; TD=Typical Development; TypSibASD=Typical Sibling of ASD Proband.

© 2023 Pierce K et al. *JAMA Network Open*.

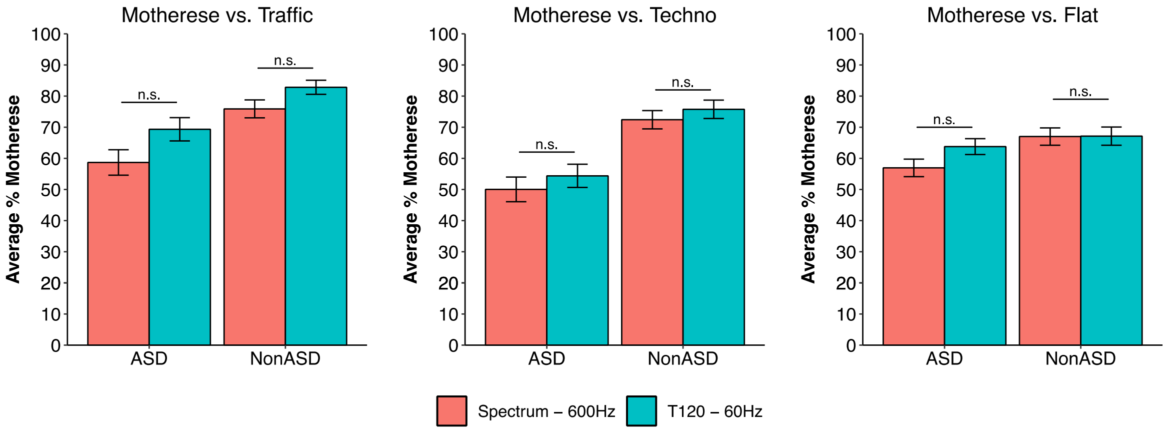

eFigure 5. Comparison of Motherese Fixation Levels Across Eye-Tracking Systems. Bar graphs illustrating comparisons in fixation levels towards motherese for the Spectrum and T120 eye tracking systems. As illustrated, average motherese fixation levels did not differ across the two machines. ASD = Autism Spectrum Disorder; NonASD = all other toddlers excluding ASD; n.s. = not significant.

© 2023 Pierce K et al. *JAMA Network Open*.

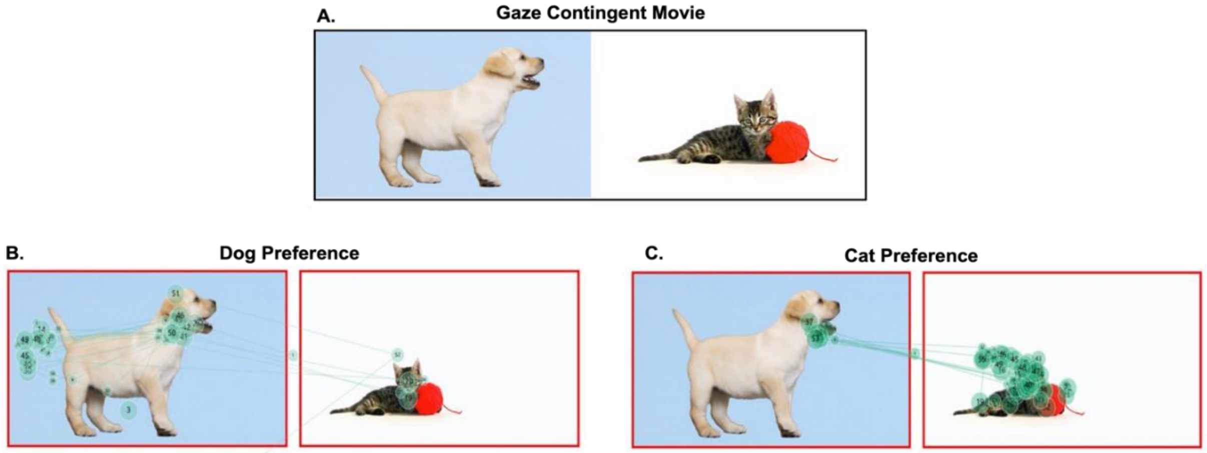

eFigure 6. Pre-Experiment Dog-Cat Training Task. A. Sample image from the Dog Cat Training Task using gaze contingent technology. If a toddler fixates on the dog he wags his tail and gently barks; if he fixates on the kitten she plays with the yarn and gently purrs. **B&C.** Fixation plots illustrating the order of fixations and clear evidence of gaze alteration during training. Numbers inside the circle represents the order that the fixation occurred. Although evidence of gaze alteration is clear, some toddlers clearly preferred to look at the dog **(B),** while other toddlers clearly preferred to look at the cat **(C).**

© 2023 Pierce K et al. *JAMA Network Open*.

e-RESULTS

Total Looking Time

For the *Motherese vs. Traffic* paradigm, the total amount of time that toddlers spent fixating on the overall movie did not differ between diagnostic groups ($F(4,581)=1.83$, $p=0.12$, *ns.*, $\eta^2=0.01$; ASD 51.10 sec ± 7.31 sec, ASD Features 53.44 sec ± 6.69 sec, Delay 51.94 sec ± 8.09 s, TD 52.68 sec ± 7.99 sec, TypSibASD 54.62 sec ± 7.99 sec).

For the *Motherese vs. Techno* paradigm, there was a significant main effect of diagnosis on total looking time ($F(4, 472)=3.74$, $p<0.05$, $\eta^2=0.03$; ASD 51.34 sec ± 7.99 sec, ASD Features 54.97 sec ± 3.69 sec, Delay 51.04 sec ± 8.52 sec, TD 54.07 sec ± 6.31 sec, TypSibASD 55.62 sec ± 3.07 sec).

For the *Motherese vs. Flat Affect* paradigm, there was a significant, main effect of diagnosis on total looking time $F(4, 381)=9.13$, $p<0.0001$****, $\eta^2=0.09$, which was driven by differences between TD and ASD toddlers (ASD 46.50 sec ± 8.49 sec, ASD Features 50.55 sec ± 7.80 sec, Delay 49.23 sec ± 8.12 sec, TD 52.41 sec ± 6.59 sec, TypSibASD 50.56 sec ± 8.12 sec; follow-up pairwise comparisons using Tukey's HSD: TD vs. ASD $p<0.001$***).

Pairwise Comparison of Diagnostic Group Differences in Motherese Fixation Levels

Pairwise comparisons of diagnostic group differences using Tukey's HSD indicate that across *Techno* and *Traffic* motherese paradigms, ASD toddlers exhibited the lowest levels of fixation to Motherese compared to almost all other diagnostic groups. Conversely, motherese fixation levels among TD toddlers was comparable to ASD Features, Delay, and TypSibASD toddlers. On the *Flat Affect* paradigm, motherese fixation levels were also lower in ASD compared to TD toddlers, but comparable to fixation levels observed for other diagnostic groups. **See eTable 1.**

Males vs. Females

Sex was a significant covariate of Motherese fixation levels but only for the *Motherese vs. Traffic* test (One-way ANCOVA, Effect of Sex: $F(1,581)=9.01$ $p<.01$, $\eta^2=0.01$) with ASD females fixating on motherese more than ASD males. Sex was not a significant covariate for the *Motherese vs. Techno* or *Motherese vs. Flat Affect* paradigms. **See eFigure 7.**

Correlations Between Motherese Paradigms

© 2023 Pierce K et al. *JAMA Network Open*.

There were strong correlations between motherese fixation levels obtained from the *Motherese vs. Techno* and *Motherese vs. Traffic* paradigms when toddlers were collapsed across groups (all Dx: Pearson's $r = 0.6$, $p<.0001$) or split into ASD and non-ASD groups (ASD Pearson's $r=0.57$ $p<.0001$, NonASD Pearson's $r=0.36$, $p<.0001$). Weaker correlations were observed when *Techno* and *Traffic* paradigms were compared with *Flat Affect* (all Dx: *Traffic vs. Flat* Pearson's $r=0.19$, $p<.001$, *Techno vs. Flat* Pearson's $r=0.24$, $p<.0001$). **See eFigure 8.**

Correlations Between Motherese Fixation Levels and Social and Language Ability in the *Techno* and *Traffic* Paradigms

Overall, results indicated a strong correlation between social and language abilities in the ASD group. With two minor exceptions (i.e., correlation between Mullen receptive language T score and motherese speech in the TD and Typ Sib groups during the Techno paradigm, no significant correlations were found for any of the diagnostic groups.

Differences in clinical phenotype between toddlers with Low, Middle, and High attention to Motherese speech during the Traffic paradigm

Similar to results from the *Techno* Paradigm reported in the main paper, toddlers with ASD, low middle and high levels of attention towards motherese speech were associated with different clinical profiles. **See eFigure 9.**

Relationships Between Motherese Fixation Levels and Age

Age was a significant covariate of Motherese fixation levels for both the *Motherese vs. Traffic* and *Motherese vs. Flat Affect* paradigms (One-Way ANCOVA, Effect of Age: *Traffic* $F(1,581)=22.66$ $p<.0001$, $\eta^2=0.01$; *Flat* $F(1,382)=11.27$ $p<.001$, $\eta^2=0.03$). However, upon examination of age-related changes in motherese fixation levels stratified by diagnostic groups across all paradigms, few consistent, significant relationships between age and fixation levels were observed. **See eFigure 10.**

Test-Retest Reliability

188 toddlers (117 ASD, 14 ASD-Feat, 14 Delay, 38 TD, and 5 TypSibASD) ranging in age from 12-48 months participated in a retest session within 1 hour to 24 months following their first Motherese test. Toddlers were divided into 3 test-retest categories depending on the time interval between the 1st and 2nd eye

© 2023 Pierce K et al. *JAMA Network Open*.

tracking test: short term, intermediate and long term. Note that not all toddlers in the re-test sample completed all tests. For all Motherese paradigms, short and intermediate interval retests were more highly intercorrelated than longer interval retests that occurred at least 1 year after initial eye tracking (*Motherese vs. Traffic* 0-6 months ICC=0.38 p<0.05, 6-12 months ICC=0.56 p<0.0001 vs. >12 months ICC=0.16 p=n.s.; *Motherese vs. Techno* 0-6 months ICC=0.77 p<0.0001, 6-12 months ICC=0.47 p<0.0001 vs. >12 months ICC=0.48 p<0.01 See **eTable *5*.**

© 2023 Pierce K et al. *JAMA Network Open*.

A.

Pairwise Comparison of Motherese Fixation Across Dx Groups Using Tukey's HSD (Traffic Paradigm)

DX	Comparison Dx	Mean Difference	Std. Error	Sig.	95% Confidence Interval Lower Bound	Upper Bound
ASD	ASD Features	-23.16	4.90	<0.0001****	-36.96	-9.36
	Delay	-18.35	3.20	<0.0001****	-27.36	-9.35
	TD	-20.59	2.66	<0.0001****	-28.09	-13.09
	TypSibASD	-18.48	7.41	0.08	-39.35	2.39
ASD Features	ASD	23.16	4.90	<0.0001****	9.36	36.96
	Delay	4.81	5.57	0.90	-10.89	20.50
	TD	2.57	5.28	0.99	-12.32	17.45
	TypSibASD	4.68	8.70	0.98	-19.83	29.18
Delay	ASD	18.35	3.20	<0.0001****	9.35	27.36
	ASD Features	-4.81	5.57	0.90	-20.50	10.89
	TD	-2.24	3.74	0.97	-12.78	8.30
	TypSibASD	-0.13	7.83	1.00	-22.18	21.93
TD	ASD	20.59	2.66	<0.0001****	13.09	28.09
	ASD Features	-2.57	5.28	0.99	-17.45	12.32
	Delay	2.24	3.74	0.97	-8.30	12.78
	TypSibASD	2.11	7.66	1.00	-19.46	23.68
TypSibASD	ASD	18.48	7.41	0.08	-2.39	39.35
	ASD Features	-4.68	8.70	0.98	-29.18	19.83
	Delay	0.13	7.83	1.00	-21.93	22.18
	TD	-2.11	7.66	1.00	-23.68	19.46

B.

Pairwise Comparison of Motherese Fixation Across Dx Groups Tukey's HSD (Techno Paradigm)

DX	Comparison Dx	Mean Difference	Std. Error	Sig.	95% Confidence Interval Lower Bound	Upper Bound
ASD	ASD Features	-27.38	5.23	<0.001***	-42.13	-12.62
	Delay	-21.47	3.84	<0.001***	-32.28	-10.66
	TD	-26.40	3.34	<0.001***	-35.83	-16.97
	TypSibASD	-33.63	8.25	<0.001***	-56.91	-10.36
ASD Features	ASD	27.38	5.23	<0.001***	12.62	42.13
	Delay	5.91	6.15	0.86	-11.43	23.25
	TD	0.98	5.86	1.00	-15.54	17.49
	TypSibASD	-6.26	9.55	0.96	-33.18	20.67
Delay	ASD	21.47	3.84	<0.001***	10.66	32.28
	ASD Features	-5.91	6.15	0.86	-23.25	11.43
	TD	-4.93	4.63	0.81	-17.98	8.12
	TypSibASD	-12.16	8.83	0.62	-37.08	12.75
TD	ASD	26.40	3.34	<0.001***	16.97	35.83
	ASD Features	-0.98	5.86	1.00	-17.49	15.54
	Delay	4.93	4.63	0.81	-8.12	17.98
	TypSibASD	-7.23	8.64	0.91	-31.61	17.14
TypSibASD	ASD	33.63	8.25	<0.001***	10.36	56.91
	ASD Features	6.26	9.55	0.96	-20.67	33.18
	Delay	12.16	8.83	0.62	-12.75	37.08
	TD	7.23	8.64	0.91	-17.14	31.61

C.

Pairwise Comparison of Motherese Fixation Across Dx Groups Tukey's HSD (Flat Affect Paradigm)

DX	Comparison Dx	Mean Difference	Std. Error	Sig.	95% Confidence Interval Lower Bound	Upper Bound
ASD	ASD Features	-1.37	3.65	1.00	-11.75	8.88
	Delay	-3.89	2.75	0.59	-11.71	3.82
	TD	-7.29	2.17	<0.01**	-13.49	-1.22
	TypSibASD	-6.13	6.54	0.87	-24.66	12.30
ASD Features	ASD	1.37	3.65	1.00	-8.88	11.75
	Delay	-2.52	4.29	0.97	-14.63	9.61
	TD	-5.92	3.93	0.53	-17.02	5.19
	TypSibASD	-4.76	7.32	0.96	-25.42	15.94
Delay	ASD	3.89	2.75	0.59	-3.82	11.71
	ASD Features	2.52	4.29	0.97	-9.61	14.63
	TD	-3.40	3.12	0.79	-12.22	5.40
	TypSibASD	-2.24	6.89	0.99	-21.68	17.21
TD	ASD	7.29	2.17	<0.01**	1.22	13.49
	ASD Features	5.92	3.93	0.53	-5.19	17.02
	Delay	3.40	3.12	0.79	-5.40	12.22
	TypSibASD	1.15	6.71	1.00	-17.76	20.11
TypSibASD	ASD	6.13	6.54	0.87	-12.30	24.66
	ASD Features	4.76	7.32	0.96	-15.94	25.42
	Delay	2.24	6.89	0.99	-17.21	21.68
	TD	-1.15	6.71	1.00	-20.11	17.76

eTable 1. Pairwise Comparisons of Diagnostic Group Differences Across All Three Motherese Paradigms. Mean differences are computed based on estimated marginal means. All comparisons are reported using Tukey's HSD. ASD = autism spectrum disorders; TypSibASD = typical sibling of a toddler diagnosed with ASD; TD = typically developing. Note that given the minimal between group differences found in the Flat Affect Paradigm, this paradigm was not used in final data analyses. Data are shown here to here illustrate the reason why this paradigm was dropped.

© 2023 Pierce K et al. *JAMA Network Open*.

Cross Tabulation of Eye Tracking Test Results: % Fixation Motherese

Motherese vs. Traffic

Eye-Tracking Predicted Diagnosis	Diagnosis: ASD	Diagnosis: NonASD
ASD	**67** True Positive	**4** False Positive
NonASD	**308** False Negative	**209** True Negative

Motherese vs. Techno

Eye-Tracking Predicted Diagnosis	Diagnosis: ASD	Diagnosis: NonASD
ASD	**91** True Positive	**6** False Positive
NonASD	**226** False Negative	**156** True Negative

Motherese vs. Flat Affect

Eye-Tracking Predicted Diagnosis	Diagnosis: ASD	Diagnosis: NonASD
ASD	**10** True Positive	**3** False Positive
NonASD	**217** False Negative	**159** True Negative

Cross Tabulation of Eye Tracking Test Results: Saccades/Sec in Motherese AOI

Motherese vs. Traffic

Eye-Tracking Predicted Diagnosis	Diagnosis: ASD	Diagnosis: NonASD
ASD	**31** True Positive	**10** False Positive
NonASD	**344** False Negative	**203** True Negative

Motherese vs. Techno

Eye-Tracking Predicted Diagnosis	Diagnosis: ASD	Diagnosis: NonASD
ASD	**90** True Positive	**8** False Positive
NonASD	**227** False Negative	**154** True Negative

Motherese vs. Flat Affect

Eye-Tracking Predicted Diagnosis	Diagnosis: ASD	Diagnosis: NonASD
ASD	**11** True Positive	**8** False Positive
NonASD	**216** False Negative	**154** True Negative

Cross Tabulation of Index Test Results: % Motherese (Combined) Fixation

Motherese vs. Traffic

Predicted Diagnosis	ASD	NonASD
ASD	**97** True Positive	**8** False Positive
NonASD	**186** False Negative	**139** True Negative

eTable 2. Cross-Tabulation Tables from the *Motherese vs. Traffic*, *Motherese vs. Techno* and *Motherese vs Flat Affect* paradigms for % Fixation Motherese and # of Saccades per Second within the Motherese AOI metrics. The last table shows the results of combined % Fixation Motherese for *Motherese vs. Traffic* and *Motherese vs. Techno*: a subject has been considered as ASD if failed at least one of the motherese test. Note that given the low true positive rate found in the Flat Affect Paradigm, this paradigm was not used in final data analyses. Data are shown here to here illustrate the reason why this paradigm was dropped. ASD=Autism Spectrum Disorder; TD=Typical Development; TypSibASD=Typical Sibling of ASD Proband

Metric - % Fixation

Motherese vs Traffic
% Fixation Motherese
(Threshold ≤ 30% Fixation)

Statistic	Value	95% CI
Sensitivity	17.87%	14.12% to 22.13%
Specificity	98.12%	95.26% to 99.49%
Positive Predictive Value	94.37%	86.10% to 97.84%
Negative Predictive Value	40.43%	39.21% to 41.65%

Motherese vs Techno
% Fixation Motherese
(Threshold ≤ 30% Fixation)

Statistic	Value	95% CI
Sensitivity	28.71%	23.79% to 34.03%
Specificity	96.30%	92.11% to 98.63%
Positive Predictive Value	93.81%	87.16% to 97.13%
Negative Predictive Value	40.84%	39.01% to 42.69%

Motherese vs Flat
% Fixation Motherese
(Threshold ≤ 30% Fixation)

Statistic	Value	95% CI
Sensitivity	4.41%	2.13% to 7.95%
Specificity	98.15%	94.68% to 99.62%
Positive Predictive Value	76.92%	48.24% to 92.26%
Negative Predictive Value	42.29%	41.43% to 43.14%

Metric - # Saccades/Sec

Motherese vs Traffic
Saccades/Sec in Motherese AOI
(Threshold ≥ 2.85 saccades/sec)

Statistic	Value	95% CI
Sensitivity	8.27%	5.69% to 11.53%
Specificity	95.31%	91.54% to 97.73%
Positive Predictive Value	75.61%	60.80% to 86.11%
Negative Predictive Value	37.11%	36.12% to 38.11%

Motherese vs Techno
Saccades/Sec in Motherese AOI
(Threshold ≥ 2.47 saccades/sec)

Statistic	Value	95% CI
Sensitivity	28.39%	23.49% to 33.70%
Specificity	95.06%	90.50% to 97.84%
Positive Predictive Value	91.84%	84.84% to 95.76%
Negative Predictive Value	40.42%	38.56% to 42.30%

Motherese vs Flat
Saccades/Sec in Motherese AOI
(Threshold ≥ 3.18 saccades/sec)

Statistic	Value	95% CI
Sensitivity	4.85%	2.44% to 8.50%
Specificity	95.06%	90.50% to 97.84%
Positive Predictive Value	57.89%	36.13% to 76.97%
Negative Predictive Value	41.62%	40.51% to 42.74%

Combined – Traffic and Techno Paradigms
% Fixation Motherese
(Threshold ≤ 30% Fixation)

Statistic	Value	95% CI
Sensitivity	34.28%	28.75% to 39.81%
Specificity	94.56%	90.89% to 98.23%
Positive Predictive Value	92.38%	87.50% to 97.26%
Negative Predictive Value	42.77%	40.50% to 45.38%

eTable 3. Metrics of Diagnostic Accuracy with 95% Confidence Intervals for both % Fixation on Motherese and # of Saccades per second Metrics across both the paradigms. The last table shows the results of combined % Fixation Motherese for *Motherese vs. Traffic* and *Motherese vs. Techno*: a subject has been considered as ASD if failed at least one of the motherese test. Note that given the low sensitivity and PPV in the Flat Affect Paradigm, this paradigm was not used in final data analyses. Data are shown here to here illustrate the reason why this paradigm was dropped. Sec = Second.

© 2023 Pierce K et al. *JAMA Network Open*.

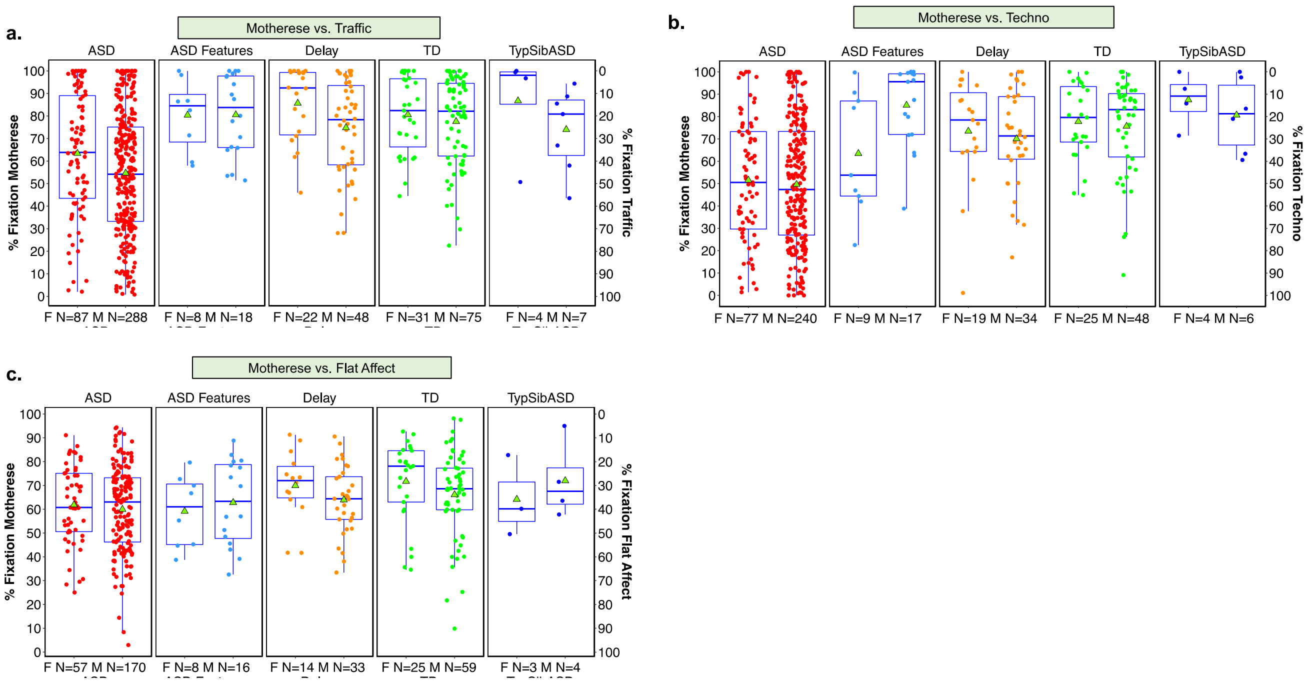

eFigure 7. Motherese Fixation Levels Stratified by Sex. ASD = Autism Spectrum Disorders, TD = typically developing, TypSibASD = typical sibling of a toddler diagnosed with ASD, M = males, F = females, N = number of subjects.

© 2023 Pierce K et al. *JAMA Network Open*.

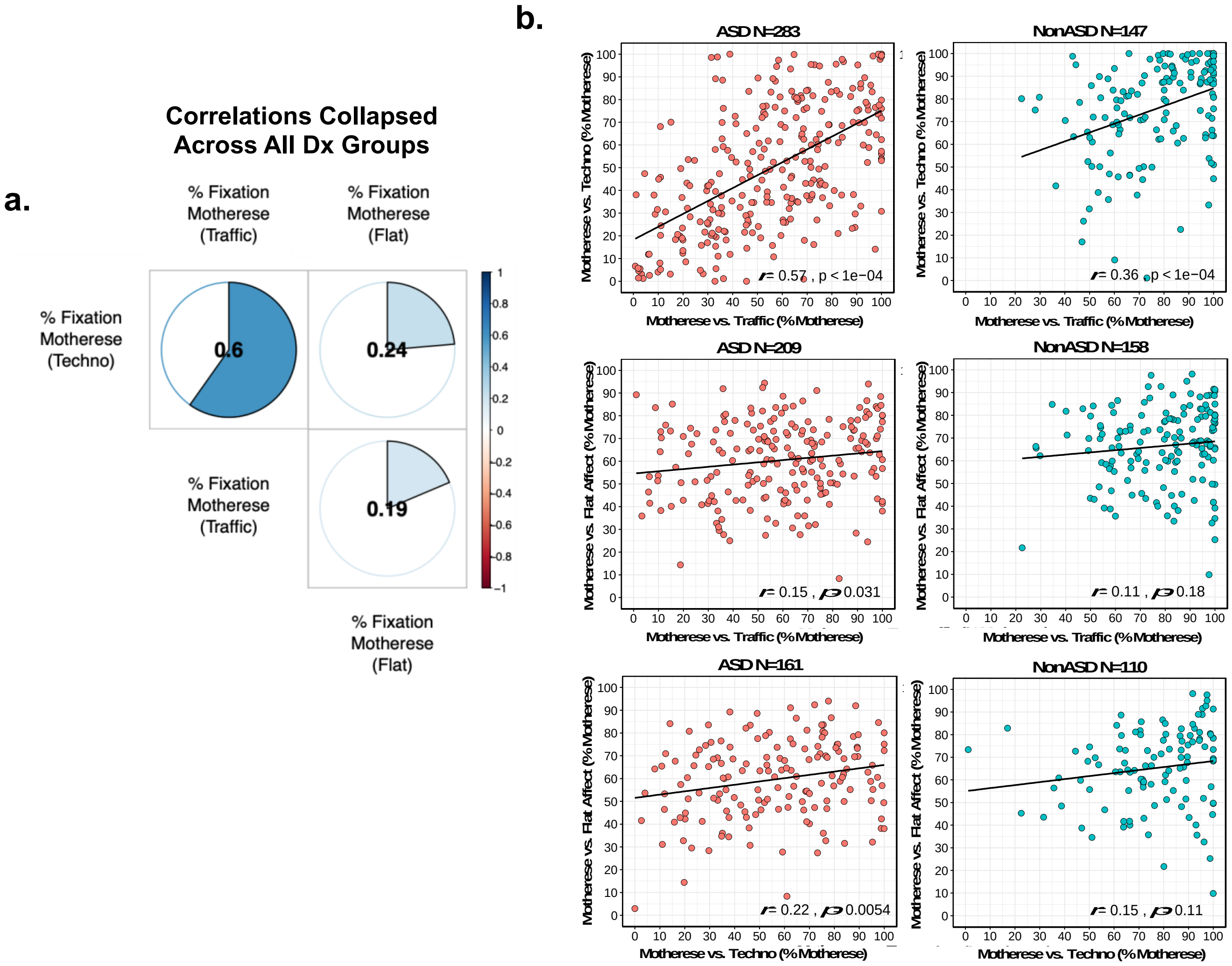

eFigure 8. Relationships Between Motherese Fixation Levels Across Motherese Paradigms. a. Strong, significant correlations were found between the *Motherese vs. Techno* and *vs. Motherese Traffic* paradigms when all subjects, independent of Dx, were considered. Motherese fixation levels in *Motherese vs. Flat* were less strongly correlated with the other tests. **b.** Motherese fixation levels have similar relationships when toddlers are stratified into ASD vs non-ASD groups. ASD = autism spectrum disorder, NonASD = toddlers of all other developmental types, excluding ASD toddlers, N = number of subjects.

© 2023 Pierce K et al. *JAMA Network Open*.

Table of Pearson's r Values Showing Relationships Between Clinical Measures and Motherese Fixation Levels (Techno Paradigm)						
	ASD	ASD Features	Delay	TD	TypSibASD	Collapsed
ADOS SA Total	-0.43****	-0.01$^{0.96}$	-0.04$^{0.76}$	-0.19$^{0.11}$	0.01$^{0.97}$	-0.52****
Vineland Socialization	0.33****	-0.44*	-0.18$^{0.21}$	0.08$^{0.49}$	-0.37$^{0.29}$	0.34****
Mullen ELT	0.44****	-0.20$^{0.32}$	0.11$^{0.43}$	0.20$^{0.09}$	-0.19$^{0.60}$	0.49****
Mullen RLT	0.48****	-0.07$^{0.74}$	0.22$^{0.11}$	0.45****	-0.65$^{0.04}$	0.55****
Mullen VRT	0.41****	-0.15$^{0.47}$	0.01$^{0.94}$	0.20$^{0.08}$	-0.03$^{0.92}$	0.47****

Table of Pearson's r Values Showing Relationships Between Clinical Measures and Motherese Fixation Levels (Traffic Paradigm)						
	ASD	ASD Features	Delay	TD	TypSibASD	Collapsed
ADOS SA Total	-0.35****	-0.03$^{0.87}$	-0.01$^{0.92}$	-0.03$^{0.78}$	-0.58$^{0.06}$	-0.45****
Vineland Socialization	0.33****	0.01$^{0.97}$	-0.01$^{0.94}$	0.00$^{0.97}$	-0.13$^{0.71}$	0.37****
Mullen ELT	0.31****	-0.30$^{0.13}$	0.03$^{0.79}$	-0.02$^{0.83}$	-0.24$^{0.48}$	0.37****
Mullen RLT	0.30****	-0.20$^{0.32}$	0.01$^{0.96}$	0.04$^{0.71}$	-0.36$^{0.27}$	0.39****
Mullen VRT	-0.35****	0.04$^{0.61}$	-0.01$^{0.54}$	-0.03$^{0.08}$	-0.58$^{0.75}$	0.44****

eTable 4. Correlations Between Motherese Fixation Levels and Social and Language Ability in the Techno and Traffic Paradigms. ASD=Autism Spectrum Disorder; TD=Typical Development; TypSibASD=Typical Sibling of ASD Proband, SA= Social Affect, ELT=Expressive Language T Score, RLT=Receptive Language T Score, Visual Reception T Score.

© 2023 Pierce K et al. *JAMA Network Open*.

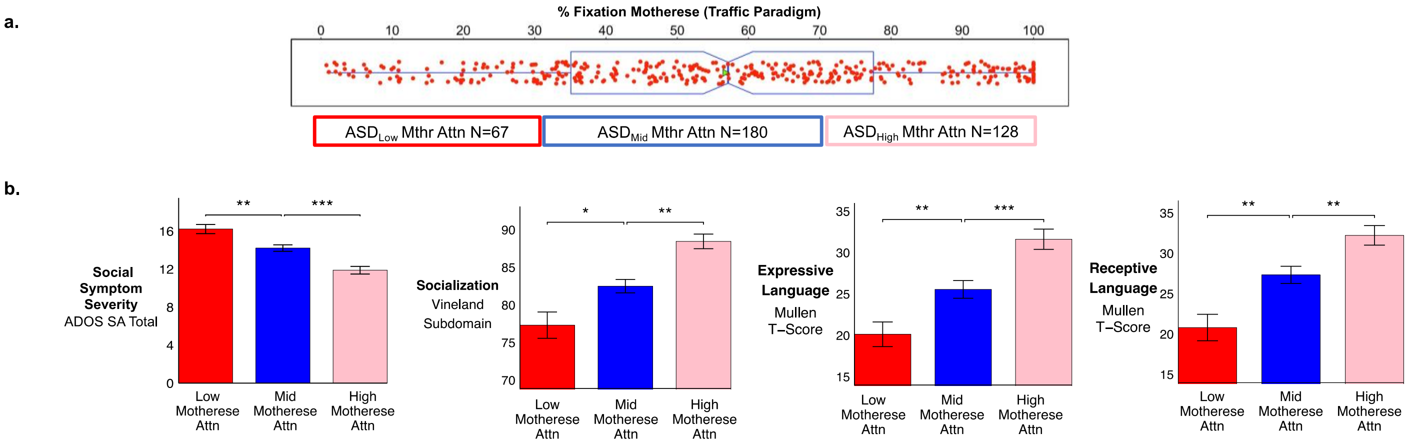

eFigure 9. Differences in Clinical Phenotype Between Toddlers with Low, Middle, and High Attention to Motherese Speech During the Traffic Paradigm. a. Stratification sample sizes and associated fixation cut off values for toddlers identified as Low, Middle or High attention towards motherese speech. **b.** Bar graphs illustrating differences in standardized test scores between subgroups. As illustrated, toddlers with ASD who displayed the lowest levels of fixation towards motherese speech (i.e., fixation levels 0-30%) showed the lowest social and language abilities as indexed by the ADOS, Vineland, and the Mullen receptive and expressive language subdomain scores. In contrast, toddlers with the highest attention levels towards motherese speech showed the strongest language and social abilities. *=p<.05; **=p<.01; ***=p<.001. Results were similar for the *Techno* paradigm as reported in the main body of the paper. Mthr=Motherese, Attn=Attention, SA=Social Affect,

© 2023 Pierce K et al. *JAMA Network Open*.

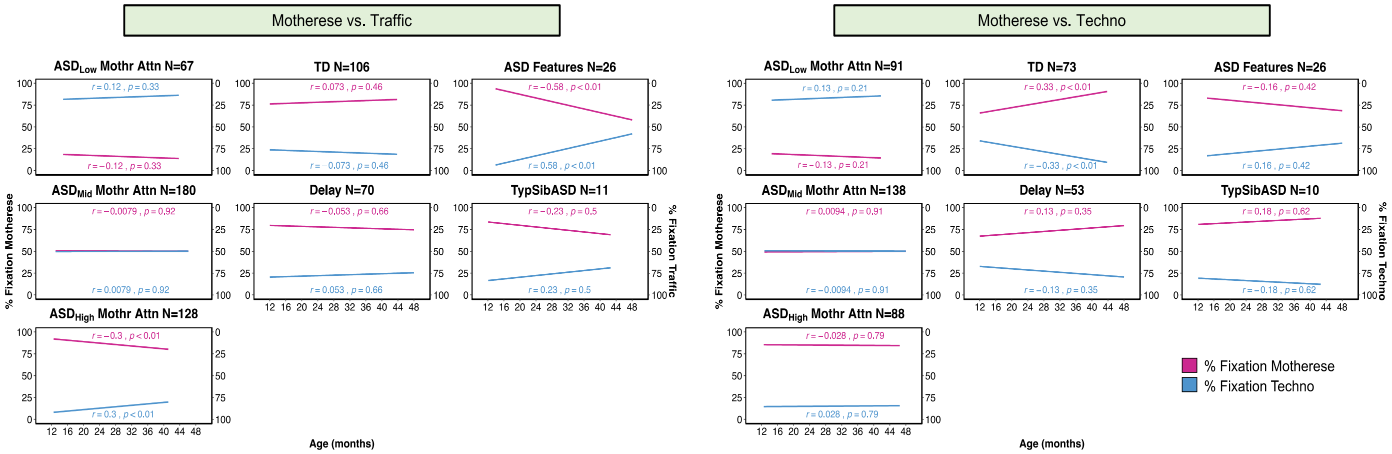

eFigure 10. Age-Related Changes in Motherese Fixation Levels. ASD = Autism Spectrum Disorders, TD = typically developing, TypSibASD = typical sibling of a toddler diagnosed with ASD, M = males, F = females, N = number of subjects, ASD_{Low} Mothr Attn = ASD subjects who fixated on Motherese for less than or equal to 30% of the time, ASD_{Mid} Mothr Attn = ASD subjects who didn't exhibit a strong preference for Motherese or non-social stimuli (Traffic or Techno), ASD_{High} Mothr Attn = ASD subjects who fixated on Motherese for at least 70% of the time.

© 2023 Pierce K et al. *JAMA Network Open*.

a.

	0-6 mo Short Term N	6-12 mo Intermediate Term N	>12 mo Long Term N
ASD	15	62	23
ASD Features	2	7	4
Delay	3	4	3
TD	7	19	8
TypSibASD	3	1	0
**Total (N)**	**30**	**93**	**38**
Absolute change score between T1 & T2, % Fixation Motherese	21.76 ± 21.63	19.11 ± 18.21	25.60 ± 19.37
Paired t-test between T1 & T2	0.24 n.s.	0.12 n.s.	***0.02****
Intraclass correlation coefficient	***0.38****	***0.56*******	0.16
95% Confidence Interval	[0.10 0.61]	[0.43 0.67]	[-0.078 0.39]
Test-Retest Reliability: Motherese vs. Traffic			

b.

	0-6 mo Short Term N	6-12 mo Intermediate Term N	>12 mo Long Term N
ASD	9	52	16
ASD Features	1	3	6
Delay	2	1	6
TD	5	9	6
TypSibASD	2	0	1
**Total (N)**	**19**	**65**	**35**
Absolute change score between T1 & T2, % Fixation Motherese	15.36 ± 14.88	22.40 ± 18.80	18.82 ± 14.72
Paired t-test between T1 & T2	0.81 n.s.	0.68 n.s.	0.72 n.s.
Intraclass correlation coefficient	***0.77*******	***0.47*******	***0.48*****
95% Confidence Interval	[0.56 0.89]	[0.29 0.61]	[0.23 0.67]
Test-Retest Reliability: Motherese vs. Techno			

eTable 5. Test-Retest Reliability. **a**. ICC values for the *Traffic* and **b**. *Techno* paradigms. T1= time point 1, T2 = time point 2, ASD = Autism Spectrum Disorder, TD = typically developing, TypSibASD = typical sibling of a toddler diagnosed with ASD, *p<0.05, **p<0.01, ****p<0.0001, n.s. = not significant.

© 2023 Pierce K et al. *JAMA Network Open*.

References

1. Pierce K, Marinero S, Hazin R, McKenna B, Barnes CC, Malige A. Eye tracking reveals abnormal visual preference for geometric images as an early biomarker of an autism spectrum disorder subtype associated with increased symptom severity. *Biological psychiatry*. 2016;79(8):657-666. doi:10.1016/j.biopsych.2015.03.032
2. Pierce K, Conant D, Hazin R, Stoner R, Desmond J. Preference for geometric patterns early in life as a risk factor for autism. *Archives of General Psychiatry*. Jan 2011;68(1):101-9. doi:10.1001/archgenpsychiatry.2010.113
3. Kwon MK, Moore A, Barnes CC, Cha D, Pierce K. Typical Levels of Eye-Region Fixation in Toddlers With Autism Spectrum Disorder Across Multiple Contexts. *Journal of the American Academy of Child and Adolescent Psychiatry*. 2019;58(10):1004-1015. doi:10.1016/j.jaac.2018.12.011
4. Moore A, Wozniak M, Yousef A, et al. The geometric preference subtype in ASD: Identifying a consistent, early-emerging phenomenon through eye tracking. *Molecular autism*. 2018;9(1):1-13. doi:10.1186/s13229-018-0202-z
5. Pierce K, Gazestani V, Bacon E, et al. Get SET Early to Identify and Treatment Refer Autism Spectrum Disorder at 1 Year and Discover Factors That Influence Early Diagnosis. *J Pediatr*. Apr 26 2021;doi:10.1016/j.jpeds.2021.04.041
6. Wetherby A, Prizant B. Communication and symbolic behavior scales developmental profile - first normed edition. Baltimore, MD: Paul H. Brookes; 2002.
7. Bujang MA, Adnan TH. Requirements for Minimum Sample Size for Sensitivity and Specificity Analysis. *J Clin Diagn Res*. Oct 2016;10(10):Ye01-ye06. doi:10.7860/jcdr/2016/18129.8744
8. Wen TH, Cheng A, Andreason C, et al. Large scale validation of an early-age eye-tracking biomarker of an autism spectrum disorder subtype. *Sci Rep*. Mar 11 2022;12(1):4253. doi:10.1038/s41598-022-08102-6
9. Lord C, Rutter M, DiLavore PC, Risi S, Gotham K, Bishop SL. *Autism Diagnostic Observation Schedule, Second Edition (ADOS-2) Manual (Part I): Modules 1-4*. Western Psychological Services; 2012.
10. Lord C, Luyster, R.J., Gotham, K. & Guthrie, W. *Autism Diagnostic Observation Schedule, Second Edition (ADOS-2) Manual (Part II): Toddler Module*. Western Psychological Services; 2012.
11. American Psychiatric Association. *Diagnostic and Statistical Manual of Mental Disorders, 5th ed (DSM-5)*. American Psychiatric Publishing; 2013.
12. Pierce K, Gazestani VH, Bacon E, et al. Evaluation of the Diagnostic Stability of the Early Autism Spectrum Disorder Phenotype in the General Population Starting at 12 Months. *JAMA Pediatr*. Jun 1 2019;173(6):578-587. doi:10.1001/jamapediatrics.2019.0624
13. Bossuyt PM, Reitsma JB, Bruns DE, et al. STARD 2015: an updated list of essential items for reporting diagnostic accuracy studies. *Bmj*. Oct 28 2015;351:h5527. doi:10.1136/bmj.h5527
14. Technology T. Accuracy and precision test method for remote eye trackers. https://stemedhub.org/resources/3311/download/Tobii_Test_Specifications_Accuracy_and_PrecisionTestMethod_version_2_1_1_.pdf
15. Shrout PE, Fleiss JL. Intraclass correlations: uses in assessing rater reliability. *Psychol Bull*. Mar 1979;86(2):420-8. doi:10.1037//0033-2909.86.2.420

© 2023 Pierce K et al. *JAMA Network Open*.
