# Supplementary material for: Level of Attention to Motherese Speech as an Early Marker of Autism Spectrum Disorder
Source: JAMA Netw Open. 2023 Feb 8;6(2):e2255125. doi: 10.1001/jamanetworkopen.2022.55125 (PMC9909502; doi:10.1001/jamanetworkopen.2022.55125)
Supplement: Supplement 2. — Data Sharing Statement [file jamanetwopen-e2255125-s002.pdf]

Data Sharing Statement

Pierce. Level of Attention to Motherese Speech as an Early Marker of Autism Spectrum Disorder. *JAMA Netw Open*. Published February 08, 2023.
doi:10.1001/jamanetworkopen.2022.55125

Data
Data available: Yes
Data types: Deidentified participant data, Data dictionary
How to access data: Data will be made available at National Institute of Mental Health data archive (NDA). https://nda.nih.gov/ Alternatively, data can be requested by emailing kpierce@health.ucsd.edu
When available: beginning date: 09-01-2023

Supporting Documents
Document types: None

Additional Information
Who can access the data: Researchers whose proposed use of the data has been approved.
Types of analyses: For any purpose
Mechanisms of data availability: Investigator must download data from NDA or obtain it directly from the lead author.
Any additional restrictions: None
